# Drug Delivery to the Posterior Segment of the Eye: Biopharmaceutic and Pharmacokinetic Considerations

**DOI:** 10.3390/pharmaceutics12030269

**Published:** 2020-03-16

**Authors:** Rubén Varela-Fernández, Victoria Díaz-Tomé, Andrea Luaces-Rodríguez, Andrea Conde-Penedo, Xurxo García-Otero, Asteria Luzardo-Álvarez, Anxo Fernández-Ferreiro, Francisco J. Otero-Espinar

**Affiliations:** 1 Department of Pharmacology, Pharmacy and Pharmaceutical Technology, University of Santiago de Compostela (USC), Campus vida, 15782 Santiago de Compostela, Spain; rubenvf1@gmail.com (R.V.-F.); victoriadiaztome@gmail.com (V.D.-T.); andrealuaces21@gmail.com (A.L.-R.); andrea.conde.penedo@rai.usc.es (A.C.-P.); xurxo.garcia.otero@gmail.com (X.G.-O.); asteriam.luzardo@usc.es (A.L.-Á.); 2 Clinical Neurosciences Group, University Clinical Hospital, Health Research Institute of Santiago de Compostela (IDIS), Travesía da Choupana s/n, 15706 Santiago de Compostela, Spain; 3 Clinical Pharmacology Group, University Clinical Hospital, Health Research Institute of Santiago de Compostela (IDIS), Travesía da Choupana s/n, 15706 Santiago de Compostela, Spain; 4 Paraquasil Group, Health Research Institute of Santiago de Compostela (IDIS), Travesía da Choupana s/n, 15706 Santiago de Compostela, Spain; 5 Molecular Imaging Group. University Clinical Hospital, Health Research Institute of Santiago de Compostela (IDIS), Travesía da Choupana s/n, 15706 Santiago de Compostela, Spain

**Keywords:** ocular pharmacokinetics, ocular drug delivery systems, ocular routes of drug administration, intravitreal administration, topical administration

## Abstract

The treatment of the posterior-segment ocular diseases, such as age-related eye diseases (AMD) or diabetic retinopathy (DR), present a challenge for ophthalmologists due to the complex anatomy and physiology of the eye. This specialized organ is composed of various static and dynamic barriers that restrict drug delivery into the target site of action. Despite numerous efforts, effective intraocular drug delivery remains unresolved and, therefore, it is highly desirable to improve the current treatments of diseases affecting the posterior cavity. This review article gives an overview of pharmacokinetic and biopharmaceutics aspects for the most commonly-used ocular administration routes (intravitreal, topical, systemic, and periocular), including information of the absorption, distribution, and elimination, as well as the benefits and limitations of each one. This article also encompasses different conventional and novel drug delivery systems designed and developed to improve drug pharmacokinetics intended for the posterior ocular segment treatment.

## 1. Introduction

**Figure 1:**
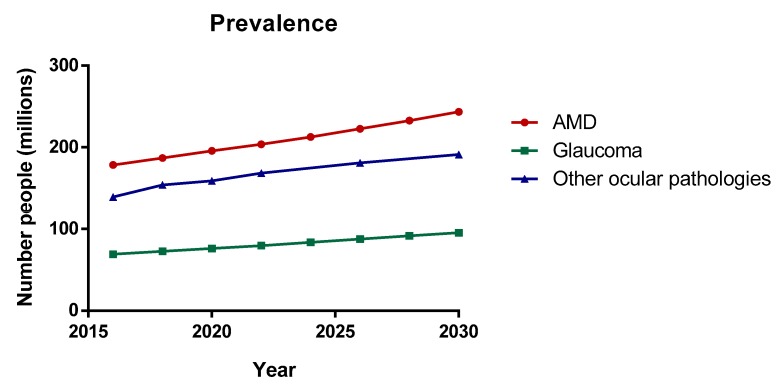
Prevalence of the main ocular pathologies. Data from the world report on vision published by the World Health Organization (WHO), 2019 [1].

The posterior segment of the eye comprises the back two-thirds of the eye, including the vitreous humor, the retina, the choroid and the optic nerve. Posterior Segment Eye Diseases (PSEDs) are then defined as the disorders that affect these tissues with the common main outcome of varying degrees of visual impartment and blindness. The most prevalent diseases are glaucoma, age-related macular degeneration (AMD) and diabetic retinopathy (DR) (see Figure 1). Nowadays, millions of people are suffering from retinal and choroid diseases and the number is increasing every year, as the incidence significantly increases with age. Both disorders are characterized by their severity and difficulty of treating. Despite numerous efforts, effective intraocular drug delivery remains unresolved and therefore, it is highly desirable to improve the current treatments of diseases affecting the vitreous cavity.

**Figure 2:**
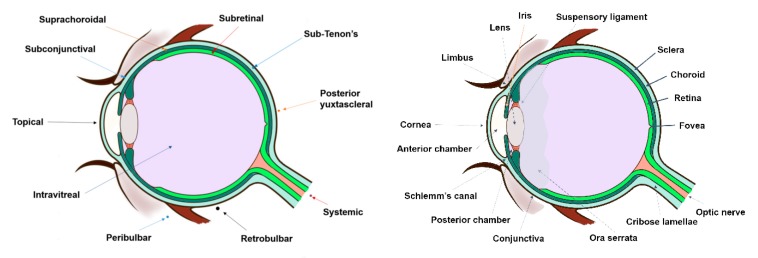
The first image represents a scheme of the different routes of drug administration to the posterior segment (dots symbolize the injection site of each route), while second image exemplifies the anatomy of the eye.

In clinical practice, the standard procedure in treating these disorders is the intravitreal administration of injected drugs, although topical and systemic administration have also been addressed with limited results. Thus, other approaches have been developed for the treatment of posterior segment diseases such as periocular, suprachoroidal, and subretinal administration (Figure 2). All these routes of drug administration consist of the drug/system injection in the surroundings of the target site. Periocular administration includes subconjunctival, sub-Tenon’s, peribulbar, retro bulbar, and posterior juxtascleral injection. However, these injections might not result in therapeutic drug levels in the target site due to the necessity of crossing several barriers to reach the intended site of action. This limitation could be overcome by using more effective drug delivery systems, where improved therapies may also be achieved.

One of the main drawbacks of drug delivery to the back of the eye is the narrow interval dosing because of the low effectiveness and bioavailability of the administered drug. Also, it should be taken into consideration that intravitreal and periocular routes are highly invasive, being associated to discomfort and patient compliance. Intraocularly, a prolonged drug release not only entails the patient acceptability by increasing their quality of life, but also, a notable reduction in the economic costs associated to the hospital stays due to the frequently repeated injections that are necessary to complete the treatment.

Drug delivery to the posterior segment of the eye still remains a great challenge in the pharmaceutical industry due to the complexity and particularity of the anatomy and physiology of the eye. Some advances have been made with the purpose of maintaining constant drug levels in the site of action. The anatomical ocular barriers have a great impact on drug pharmacokinetics and, subsequently, on the pharmacological effect. For this reason, understanding pharmacokinetics plays a critical role in the design of effective pharmaceutical forms. 

The aim of this review is to show an overview of the main aspects involved in ocular drug pharmacokinetics intended to treat PSEDs. A discussion of the different factors that are involved in the ocular drug delivery is first made, encompassing the different routes towards the posterior segment of the eye. Physiological barriers and drug transport pathways are described and the advantages and drawbacks of different administration routes to the eye are also discussed.

## 2. Routes of Drug Delivery to the Posterior Segment of the Eye

### 2.1. Intravitreal Administration

Intravitreal injection is the mainstream route of administration to treat diseases affecting the posterior segment of the eye. The drug is placed directly into the vitreous humor, though it is a highly targeted drug route. It has the inconvenience of being an invasive route. However, intravitreal drug administration is always selected to deliver to the posterior ocular segment due to the possibility of overcoming systemic exposure and obtaining high drug levels into the vitreous chamber. 

#### 2.1.1. Vitreous and Posterior Compartment Barriers to Drug Delivery

##### Vitreous

The vitreous body shows a transparent jelly-like structure mostly composed of types II, IX, and V/XI collagen fibers, whose spaces are filled with glycosaminoglycans, mainly hyaluronic acid. This structure acts as both a static and dynamic barrier. The static barrier is the vitreous structure by itself, although it is not a very restrictive barrier in terms of molecular mobility. The so-called dynamic barrier consists of the flow and clearance processes.

Initially, the superficial charge becomes vitally important for positively charged particles due to interactions with the components of the vitreous network such as hyaluronic acid (negatively charged), while negatively charged particles diffuse without any problem. Likewise, particle size may also impact on drug permeation, even though this parameter seems to be less clear. Certainly, several studies showed the distribution of same-sized particles (1 µm) through the vitreous is charge-dependent [2].

The convective flow from the anterior to the posterior segment of the eye should be taken into consideration for drug uptake due to the difference in pressure of the vitreous. This natural fact influences the drug distribution, having more impact on large molecules.

The drug particle size also plays a role in the type of clearance that drugs undergo, since smaller and more lipophilic drugs are mainly cleared in the posterior segment of the eye because of their ability of crossing the blood–retinal barrier (BRB), while larger and hydrophilic molecules are removed through anterior segment due to the aqueous humor outflow [2,3].

##### Posterior Compartment Barriers to Drug Delivery

1. Inner Limiting Membrane

The inner limiting membrane is a mechanical and electrostatic barrier located between the vitreous and the retina composed of collagen, laminin, and fibronectin. Its composition and thickness (~4 µm) may be variable along its structure, showing a 10 nm pore size.

Both drug charge and size can play an important role in the passage of molecules through this membrane, being the charge the most critical factor. Thus, positively charged nanoparticles seem to present more difficulty than neutral and negatively charged ones because of the negative charge of the membrane [3,4].

2. Neural Retina

**Figure 3:**
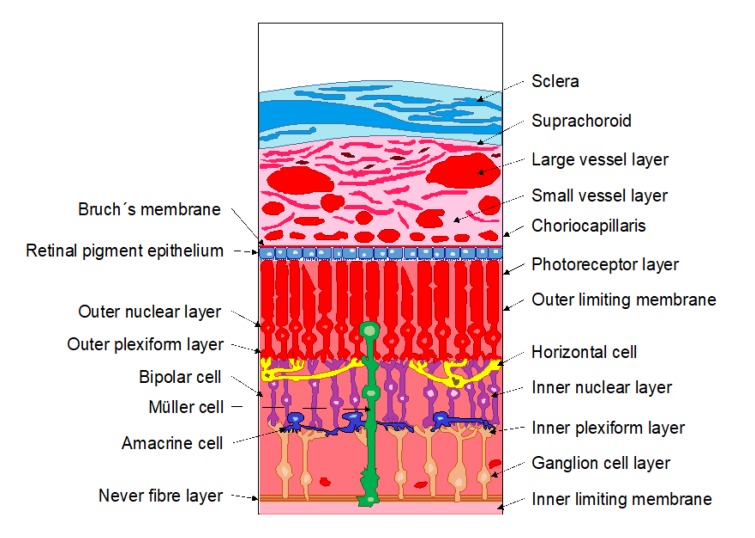
Schematic drawing of the sclera, choroid, and retina.

The neural retina is the innermost layer of the eye and consists of a multi-layered structure which is responsible for transmitting the light to the brain by means of photoreceptors, neurons, and glial cells (Figure 3). These layers are: The outer limiting barrier, the outer nuclear layer, the outer plexiform layer, the inner nuclear layer, the inner plexiform layer, the ganglion cell layer, and the nerve fiber layer. It also must be taken into account that molecules cannot freely diffuse through the retina due to Muller glial cells presence between the inner and the outer limiting membranes. Furthermore, certain components of the retina can interact with some molecules, hindering their diffusion [2,3].

3. Blood–Retinal Barrier (BRB)

The BRB is composed of two types of cells: The retinal capillary endothelial cells (which make up the inner BRB) and the retinal pigment epithelial cells (RPE) (which form the outer BRB) [5]. Both are considered an important impediment to retinal drug delivery as it restricts the drug transport between neural retina and circulating blood [4]. In the RPE, drug permeability is determined by physicochemical factors (e.g., molecular weight, lipophilicity, protein binding), as well as drug concentration gradient and their affinity with the existing transporters at that level, which can increase or decrease drug transfer through RPE [6].

The BRB limits the drug diffusion from the vitreous humor to inner parts of the retina. It is composed of the inner BRB, which contacts with the vitreous humor and it is formed by capillary endothelial cells connected by tight junctions, and the outer BRB, also called the RPE, which exhibits a high amount of melanin, being surrounded by choroidal capillaries [3,7,8]. 

Paracellular transport through retinal capillaries is limited by the tight junctions (about 2 nm), although certain larger-size molecules can transcellularly permeate by passive diffusion and/or active transport (e.g., ganciclovir or dexamethasone) [9,10]. Nevertheless, both processes are also restricted due to the absence of fenestrations and the lack of transport vesicles in the endothelial cells.

The outer BRB located between the photoreceptors and the choriocapillaris, is composed of tight, gap and adherent junctions that can be removed by molecules via passive diffusion. Also, the pump proteins that are in the layer and the invagination vesicles allow active transport (e.g., Na^+^/K^+^-ATPase, P-glycoprotein, Multidrug Resistance Proteins -MRP1-) [11].

Molecules permeation is restricted depending on their size, charge, and/or lipophilicity. Thus, hydrophilic compounds follow the paracellular route through tight junctions while lipophilic molecules can cross the epithelium by the transcellular route. It has been demonstrated that only small lipophilic molecules can easily diffuse from bloodstream to the vitreous and vice versa [2,3,12].

4. Choroid

The choroid is a highly vascularized barrier that lies between the RPE and the sclera (Figure 3). This layer supplies oxygen and nutrients to the retina. It has a 200 µm thickness and it is divided into five layers: The Bruch’s membrane, the choriocapillaris layer, two vascular layers, and the suprachoroidal layer [2]. 

The Brunch´s membrane presents a 2–4 µm thickness, whose structure is composed of collagen and elastin fibers. The choriocapillaris layer is formed by highly fenestrated capillaries with a 6–12 nm pore size, allowing the passage of large molecules [13]. The suprachoroid is located between the sclera and the choroid and it is composed of collagen fibers, melanocytes, and fibroblasts. 

In terms of drug delivery, it must be taken into consideration that choroid shows two different behaviors: (1) It acts as a static barrier due to suprachoroid structure and (2) it provides a dynamic barrier as a consequence of a high choriocapillaris-layer blood flow. Both actions prevent the passage of hydrophilic compounds while positively charged lipophilic drugs can stabilize bindings with the tissue, leading to slow-release depots. The molecular size of the drug also determines the diffusivity into the posterior segment [2,12].

5. Sclera

The sclera is the outer opaque layer of the eye (Figure 3), which provides support to this structure. It shows a 0.5–1 mm average thickness, although it varies along the eye. It is composed of collagen fibers, proteoglycans, and glycoproteins, creating a network in an aqueous medium [14,15]. The organization of the collagen fibers gives rise to different regions, such as: Tenon’s capsule, episcleral layer, the scleral stroma tissue and the lamina fusca, melding into the underlying choroid.

Drug permeability through the sclera depends on molecular weight, molecular radius, charge and lipophilicity, although it is usually more permeable than the cornea and conjunctiva [2,12]. Large molecules, up to 150 kDa, find harder to get through the sclera and their clearance is lower than smaller molecules. Transscleral diffusion consists of the permeation through porous spaces within a collagen aqueous network. Likewise, the hydrophilic molecules (e.g., methazolamide) are able to go through the sclera [16,17]. Matrix structure of proteoglycans is negatively charged at physiological pH and, consequently, the passage of negatively charged solutes across the sclera is facilitated [18].

#### 2.1.2. Advantages and Limitations

Intravitreal administration is performed by injecting a drug solution, suspension, and even intraocular implants into the vitreous cavity. In recent years, the intravitreal injection has become the mainstream of the treatment of ocular diseases affecting the posterior segment. Direct intravitreal injection has the obvious advantage of achieving immediate therapeutic concentrations in the vitreous humor. However, sites of action are normally the retina or the choroid, so the drug is injected in the surroundings of the site of action, but it has yet to overcome some barriers in order to achieve its target site. Therefore, drug delivery into the vitreous humor does not necessarily implies that the drug will be able to exert the pharmacological effect [19]. Nevertheless, intravitreal administration has proved to be the best administration route to achieve high and effective drug concentrations in the posterior segment of the eye. 

Additionally, intravitreal administration has the advantage that largely avoids the systemic exposure; hence, the systemic adverse effects are limited. However, not all side effects are completely eliminated, being the most common ones: Endophthalmitis, vitreous detachment, retinal hemorrhage, inflammation, increased intraocular pressure, conjunctival hemorrhage, retinal toxicity, and cataracts, among others [19]. As an invasive procedure, the injection itself may cause a patient’s discomfort and eye pain, mainly where repeated injections are needed. The need to treat the posterior segment of the eye increases interest in getting available novel controlled release formulations to maintain the therapeutic drug concentration in the vitreous as well as minimizing the number of injections.

The vitreous humor is composed of a polysaccharide aqueous meshwork. Apparently, it would seem that it acts itself as a retention compartment against drug distribution [7]. Nevertheless, drugs are quickly removed from the vitreous via the anterior route to the aqueous humor, hampering prolonged release phenomena [20]. In addition, the relatively high volume of the vitreous chamber allows administering drug delivery systems of different size and form (implants and inserts). 

In clinical practice, intravitreal injections are directly administered into the vitreous cavity and the procedure is performed by a trained specialist. As an invasive procedure, some risks may be taken into account and several precautions should be taken during the intravitreal administration, such as avoiding touching the retina or the lens [19]; the vision line must be left untouched (the vision field can be blocked); and the injection incision should be as small as possible since it can prevent the development of eye infections and the leakage of vitreous [19]. Other major risks after intravitreal injection (although uncommon) are eye infections or vitreous hemorrhage. Depending on the disease being treated, a follow-up visit to the specialist should be scheduled for the administration of repeated intravitreal injections, always needed in chronic disorders such as DP or AMD [21].

Despite the associated complications to the injection procedure and discomfort after administration, repeated intravitreal injections are the only alternative to administer drugs as anti-VEGF, steroids, and antibiotics to treat pathologies affecting the posterior segment of the eye.

#### 2.1.3. Pharmacokinetics

Nowadays, the pharmacokinetics and pharmacodynamics of pharmacological agents after intravitreal administration remain relatively unexplored and poorly understood. The vitreous-humor clearance mechanisms of intraocular-administered drugs limit the duration of their effect and injection’s repeated administration. Nevertheless, there remains a real need to develop novel drug delivery systems intended to sustain release and more effective therapy. 

Pharmacokinetics studies of intravitreal injection have demonstrated the drawbacks of an invasive administration route and the difficulty of acquiring direct samples. Most of the in vivo studies have been performed in rabbits as an experimental animal model [22]. Vitreous humor samples collection may require animal sacrifice, even though novel techniques such as micro-dialysis can be applied for continuous sample collections without animal detriment [23]. According to the 3Rs regulatory frameworks, the alternative is to extract aqueous humor or blood samples and then make a correlation with the drug concentration in the vitreous humor. 

The drug concentration at any certain time after injection depends on the drug distribution volume, the initial injected dose and the elimination rate. Therefore, the two factors that predominantly affect the pharmacokinetics of injected drugs into the vitreous are their vitreous humor distribution and their clearance.

##### Drug Distribution in the Vitreous Humor

Drug distribution in the posterior segment of the eye is a crucial step in ocular pathologies treatment in order to obtain a pharmacological effect at the right target site. The factors that affect the drug distribution in the vitreous humor are predominantly its diffusion through the vitreous, the effect of the convective flow and the possible drug interactions with the vitreous humor elements. Moreover, other ocular barriers may limit the drug passage depending on the target site within the vitreous cavity [24]. 

Another aspect that should be taken into consideration is the effect of the injection itself, as the needle creates a channel through the vitreous humor. After the needle is being removed, it leaves behind a low resistance pathway for the passage of the drugs due to a change of the integrity of the vitreous humor [25]. 

The impact of both diffusion and convection flow in the drug pharmacokinetics within the vitreous humor has been studied based on different experimental data and mathematical models [7,26,27,28,29].

1. Diffusion

Once the drug is injected into the vitreous humor, it will diffuse through the vitreous humor until reaching its target tissue. The diffusion speed of this process depends on the drug physiochemical properties and the vitreous retention effect. The mesh size of the bovine vitreous humor was calculated to be approximately 500 nm [30]. Also, the different structural components presented in the vitreous constitute a barrier to the diffusion of the drugs depending of the characteristics on the drug.

Drug diffusion through the vitreous humor is created as drug concentration gradients from the injection site, which provides the moving force for the drug-particles’ movement until a concentration balance is reached, where not more diffusion occurs. Regarding the drug physicochemical properties, the molecular weight and the drug net’s charge are the parameters that most influence in the diffusion speed through the vitreous humor. 

Generally, low molecular-weight drugs (e.g., fluorescein, glycerol, mannitol) exhibit high diffusion coefficients as they do not have the diffusion restriction through the vitreous meshwork [31,32]. In fact, the diffusivity in an aqueous solution could be a specific representation of this parameter in the vitreous humor. Diffusion of high molecular-weight molecules (e.g., FITC dextran) can be limited by the vitreous structure [33].

On the other hand, the drug net’s charge could affect its diffusion since the vitreous humor is a negatively charged polymer network. Anionic and neutral molecules will not show any type of restriction in the vitreous humor considering their charge. However, cationic drugs could electrostatically interact with the negative charges of the vitreous humor [30,34]. This will lead to a decrease in the drug diffusion due to its retention in the vitreous humor.

2. Convection

Convection is the process of moving a fraction of volume of the aqueous humor produced by the ciliary processes throughout the vitreous humor towards the retina [7,26,28,29]. This process takes place due to the differences in pressure and temperature between the anterior chamber and the retinal surface [7].

The significance of the vitreous outflow effect on drug distribution depends on drug diffusivity in the vitreous [28]. It has been seen that drugs with high diffusivity values within the vitreous are not affected by the convective flow due to the high net movement through the vitreous humor, while low molecular weight drugs do not suffer any type of restriction through the vitreous. On the other hand, the relevance of the convective flow in the movement of drugs with low diffusivity values is quite unclear, even though it is generally accepted that the convective flow has a major effect when the diffusivity is lower and the flow is increased. 

Park et al. have calculated the drug diffusivity values that could be affected by the convective flow, using a mathematical model. The convection does not affect the drug distribution with high diffusivity values within the vitreous (1 × 10^−5^ cm^2^/s) but it starts to become relevant in the case of drugs with low diffusivity values (1 × 10^−5^ cm^2^/s), particularly if the flow is increased [26,28]. Moreover, Xu et al. predicted that convection could be responsible for the 30% of the drug movement through the vitreous humor [27]. In the light of these results, it can be concluded that the convection is not a major contributing factor in the drug distribution through the vitreous humor [3].

Since the intravitreal convective flow depends on pressure, it seems obvious that an increase in the intraocular pressure could change flow values in the vitreous humor. As an example, in some pathologies such as glaucoma and retinal detachment, where higher intraocular pressure values are noticed, an increase in the convection has been observed [26,29].

3. Drug Interaction with Vitreous Humor

Hyaluronic acid, as one of the main vitreous components (65–400 mg/mL concentration range), may establish charge interactions with intraocularly administered drugs that are positively charged [35]. This binding could importantly decrease the cationic-drug diffusion through the vitreous humor (e.g., poly-l-lysine) [3]. However, up to now, the effect of this binding on drug pharmacokinetics remains to be elucidated. 

4. Drug Interaction with Proteins

Protein concentration in the vitreous humor was estimated to be 4.7 ± 1.2 μg/μL in human samples [36], being the albumin the most abundant one (60–70% of the total protein) [37]. Between 1000 and 2500 different proteins have been identified in the vitreous cavity [36,38]. Although their concentration is very low in comparison with plasma concentration, the interaction between drugs and vitreous proteins can occur in the same way with plasma proteins. This interaction will lead to a free drug decrease to exert a pharmacological effect. Furthermore, the drug binding to a protein could slow down its diffusion through the vitreous humor increasing the residence time in the vitreous. 

In some vitreoretinal pathologies, such as diabetic vitreoretinopathy, an increment in protein concentration in the vitreous humor is observed, as well as new proteins are expressed and linked to inflammation and immunity processes [36]. However, there is no available data regarding the impact of the increased protein concentration in the drug interaction with the proteins in these chronic diseases.

5. Liquefaction

One factor influencing both drug diffusion and convection processes in the vitreous humor is its own liquefaction, that is, the degeneration process of the vitreous humor associated with aging. The vitreous humor is observed in both liquid and jelly forms at the same time in the vitreous cavity [39]. Nevertheless, the proportion between these two forms gets modified with age, being observed as an increase in the liquid proportion and a decrease in the gel one [7], due to the disruption of the fibers mesh composing the vitreous humor. Vitreous liquefaction could cause an increase in the drug diffusivity, particularly in those that showed a limited diffusion, since the fibers mesh presents less movement restriction of molecules in its interior. This expanded diffusion can lead to an elimination increase, although the liquefaction itself does not directly affect the drug elimination from the posterior segment of the eye. The vitreous humor higher liquefaction, the more closely the diffusivity of the molecules in it approximates to their diffusion in water [3]. On the other hand, liquefaction and the vitreous homogeneity loss through aging are also associated with an increase in the convection flow [40].

Such data entails that the treatment of different-age-group patients with the same dosing scheme might be inappropriate, leading to overdose or insufficient dosage situations. Even though, so far it is not clear whether the vitreous liquefaction can affect the intravitreal pharmacokinetics or not [41,42].

It is also worthwhile to mention that the injection position during the administration and the injected volume may have an influence on the distribution of molecules in the vitreous humor. Different injection sites affect the drug distribution and permeability through the retina [43], whereas the injected volume could affect the drug elimination in different degree, depending of the injection position [43].

On the other hand, special patient situations, such as vitrectomy (procedure where the vitreous humor is removed), also determine the drug behavior into the vitreous. The motivations to perform a vitrectomy are varied, where the most common ones are: (1) Removal of infection, (2) obtention of a better access to the retina, (3) removal of scar tissue, and (4) correction of retinal detachment.

After the vitrectomy procedure, the drug elimination is greatly increased, regardless of the elimination pathway. In different animal studies, the half-life reduction of intravitreal drugs has been detected after a vitrectomy was performed, including: Amikacin [44], amphotericin B [45,46], cefazolin [47], ceftazidime [48], ciprofloxacin [49], and vancomycin [50], as well as some biologics, such as bevacizumab [51,52], ranibizumab [51,53], and aflibercept [53]. It also must be taken into account that these studies were performed in animal models and that its extrapolation to humans lacks of enough knowledge. However, it seems to be a general decrease in the half-life in humans and might affect the efficacy of the intravitreal drug, although this aspect is still under investigation [54].

Moreover, a higher risk of retinal toxicity is expected in vitrectomized eyes. The anti-infective is supposed to be placed in contact to the retinal surface on a high concentration, instead of being distributed all over the vitreous humor, consequently causing the retinal toxicity [55]. This is extremely important in drugs as amikacin since it has proven to produce retinal toxicity [56].

Surgical vitrectomy is normally followed by the filling of the vitreous chamber with silicone oil, which acts as a long-term buffer in the management of vitreous detachment. Injected silicone oil can also behave as a slow-release reservoir for some drugs [7], although it must be taken into consideration that most anti-inflammatory drugs are not soluble in the silicone oils. Several studies confirmed the drug injection into silicone oil-filled eyes led to its migration to the aqueous interface, resulting in an increase in the local concentration and the ulterior precipitation. This may cause retinal toxicity [57,58], supposedly caused by a decrease on the preretinal-space, which can also affect the drug distribution and half-life.

Therefore, the drug pharmacokinetics in silicone oil-filled eyes is still not well defined, although some authors recommend a substantial reduction of the drug dose (1/4–1/10 of the standard dose) to prevent these phenomena [59].

##### Drug Distribution to Surrounding Tissues

Depending on the type of disease, the target site could be located in the vitreous humor itself (e.g., infections), the retina (e.g., AMD, diabetic macular degeneration), or the choroid (e.g., serpiginous choroiditis). Therefore, the drug distribution to the surrounding tissues could be considered as part of the elimination process if the site of action is in the vitreous humor or as part of the drug distribution in order to achieve its target site. Nevertheless, studies performed in rabbit eyes have shown that the distribution volume is close to the anatomical volume of the vitreous chamber which implies that the surrounding tissues do not contribute too much to the distribution process [3].

The vitreous body is limited by the anterior blood–aqueous barrier (BAB) and posterior BRB. The inner BRB allows for the permeation of small molecules, where molecules with a size higher than 2 nm are prevented from their diffusion. Likewise, the RPE is a tight cellular layer between the photoreceptors and the choroid, which its permeability depends on the molecule’s size and lipophilicity [3]. Once the drug reaches to the choroid, the drug diffusion is quite rapid, because of the higher permeability of the choroid and, subsequently, the drug is quickly removed to the blood circulation [3].

There is evidence of BRB influx-and-efflux carriers that ensure the retina is constantly supplied of nutrients and ions [7,8,60]. The evidence of efflux transporters at the BRB has been recently investigated, as the studies performed in animal models might not correlate with the results that could be obtained in humans. MDR1, BCRP, some MRP, and OATP are some of the main carrier families that have been detected in the BRB [61].

It should be noted that some drugs can be substrates of the BRB active transporters, but their contribution to the drug pharmacokinetics is still unclear. Firstly, it is needed to be addressed that the presence of active transport at the BRB could be an advantage if the drug target is in the choroid, as it will help the drug to reach the target site or even ensure that some drugs, that normally are not able to cross the BRB, can achieve the choroid. Conversely, this fact could be a disadvantage if the target site is prior to the retina or in the retina itself, the active transport will act as an elimination pathway. 

Overall, the active transport contribution on the drug movement through the BRB is quite low, the effect being also reduced over the time. As the drug concentration at the vitreous humor is usually very high after administration, the transporters are prone to be saturated [61].

The drug elimination from the vitreous humor involves the drug possible metabolism in the ocular tissues and the removal from the ocular compartments to the systemic blood flow.

Drug metabolism in the vitreous humor has not been deeply investigated. Mainly, studies have aimed at the enzyme identification in the vitreous humor, but not at analyzing its impact of drug pharmacokinetics [45]. For example, the presence of enzymes such as esterases or peptidases in rabbits´ vitreous humor should be mentioned here [62]. The drug in situ metabolism have been exploited for the development of prodrugs, such as ganciclovir esters (prodrugs with no pharmacological activity) which are biotransformed into ganciclovir (drug with pharmacological activity) once injected into the vitreous humor [62]. 

Metabolic enzymes have been detected in other ocular tissues posterior to the elimination of the drug from the vitreous humor, such as retina, ciliary body, and iris [63].

**Figure 4:**
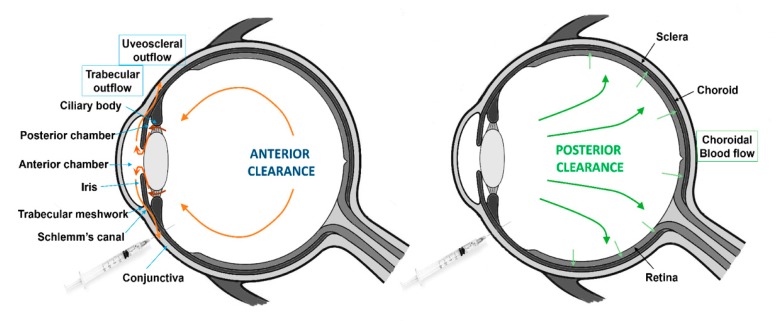
Schematic representation of the anterior and posterior clearance from the vitreous humor.

There are two major routes of drug elimination from the vitreous: Anterior and posterior clearance (Figure 4).

1. Anterior Route

After intravitreal injection, the drugs will be eliminated following the anterior route from the vitreous by a diffusion process across the lens and the ciliary body, to enter afterwards into the posterior chamber. From there, drugs are removed through the aqueous humor turnover to the anterior chamber, where they are subsequently removed along with aqueous humor by the trabecular and uveoscleral outflow [8]. The rapid turnover of the aqueous humor into the anterior chamber is the main force for the anterior clearance [7].

This elimination route is accessible for all type of drugs as they can freely move across the hyaloid membrane, avoiding the lens. However, drugs that are typically removed from this pathway are hydrophilic and large molecules that are not able to cross the retina [7]. The elimination of high molecular-weight compounds by the anterior route has been widely studied [40,42,64] (see Table 1). In fact, there is an inverse relationship between the molecular weight and the elimination rate from the vitreous.

Experimental data have determined that drugs which are removed from the anterior pathway exhibit higher half-lives than the ones that are removed from the posterior route. The relationship between vitreous half-life and aqueous humor/vitreous humor ratio is not broadly clarified. However, it is known that the presence of the drug is higher in the aqueous humor, i.e., it is removed by the anterior route, as the half-life is higher [65].

2. Posterior Route

In the posterior route, administered drugs permeate through the retina and subsequently are cleared by the choroidal blood flow. Drugs that are removed by the posterior route exhibit short half-lives due to the large surface area available for permeation and the presence of active transport mechanisms [65]. 

In posterior elimination processes, a relationship between the drug physicochemical properties and their half-lives within the vitreous humor has been identified. Durairaj et al. established that the drug molecular weight, its lipophilia, and the dose/solubility ratio at pH 7.4 are the major parameters that affect the drug half-life in the vitreous [70]. 

Therefore, the posterior route is the main elimination pathway for small and lipophilic molecules since they can easily cross the retina. The diffusion process could take place via the paracellular and/or transcellular route.

As can be seen in Table 2, some important differences are shown between the parameters affecting the anterior and posterior elimination route of drugs from the vitreous humor. 

#### 2.1.4. Drug Delivery Systems

Several reviews about the development of drug delivery systems have been published previously [71,72,73,74]. For this reason, in this article is not going to be treated in depth.

Biodegradable implants [75], non-biodegradable implants [76], biodegradable microspheres [71], nanoparticles [77], dendrimers [78], and hydrogels [79] have been used for intravitreal drug administration. Moreover, some sophisticated systems have been developed for the treatment of chronic and refractory ocular diseases, such as a microelectromechanical systems (MicroPump) [80] and a port delivery systems (PDS) [81].

In addition, to the current research on new systems of intravitreal release [82], there are already commercialized formulations such as Lucentis^®^, Ozurdex^®^, Eylea^®^, Avastyn^®^, among others (see Table 3).

### 2.2. Topical Administration

Ophthalmic topical administration by eye drops is commonly used for the treatment of anterior-segment diseases [98,99]. Most of the topically applied drugs are intended for the treatment of diseases that affect different layers of the cornea, the conjunctiva, iris, or the ciliary body [5]. However, topical administration for the treatment of posterior ocular diseases is considered an ineffective pharmacological strategy since therapeutic drug concentrations are not reached in the posterior segment of the eye due to low drug penetration. 

#### 2.2.1. Ocular Barriers for the Entry of Drugs: Precorneal Factors

After topical eye-drops administration, the first tissue barrier that drug molecules must overcome to access the target is the tear drainage of the excess volume through the nasolacrimal duct. In normal conditions, this drainage occurs at 1.45 μL·min^−1^ and it results in a drug loss into systemic circulation, especially related to hydrophilic molecules [100]. In fact, the loss of eye drop solution occurs until the tear volume returns to a normal range (7–9 µL). 

Likewise, the thin precorneal tear film secreted by different glands and the Globet cells, which is about 8 µm thickness and with a 7 µL volume, also acts as a barrier in terms of drug absorption. It is composed of three layers: mucin, an aqueous and a lipid layer. The rate of drug elimination from the tear fluid is in the range of 0.5–1.0 μL·min^−1^ [101,102]. As a result of these facts and the systemic absorption through the conjunctiva, the ocular drug absorption is limited to less than 5% (<5%) when this delivery method is used [102].

#### 2.2.2. Corneal and Anterior Compartment Barriers

##### Cornea

**Figure 5:**
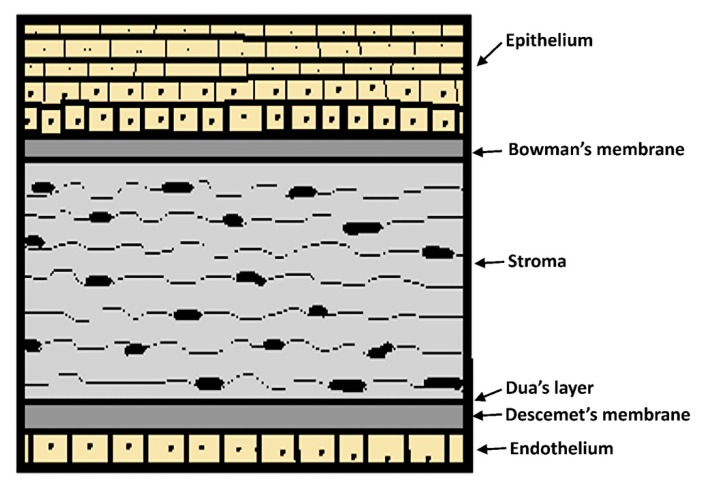
Schematic representation of the human corneal layers.

The cornea is the transparent portion surrounding the sixth anterior part of the eyeball with a 0.5 mm thickness and a 12 mm diameter. Tear film and aqueous humor provide nourishment and oxygen as it lacks blood vessels. The cornea consists of a collagen structure organized in six layers: Epithelium, Bowman’s membrane, stroma, Dua´s layer, Descemet’s membrane, and endothelium (Figure 5). The stratified, squamous and non-keratinized epithelium is the most critical barrier to penetration with a 10^−7^–10^−5^ cm^−1^ drug permeability rate because of the fact that tight junctions impair the permeation of low lipophilic molecules [12,100,103].

Drug absorption depends on their physicochemical characteristics. In consequence, only hydrophilic molecules can diffuse through the stroma due to the hydrophilic nature of the hydrated collagen. Only small molecules with a log D value between 2 and 3 can penetrate through all the layers. The paracellular route allows molecules with a 500 Da molecular weight or a <5.5 Å size penetrate across tight junctions. The solute charge is also an important factor, as the cornea decreases the absorption of negatively charged molecules because of the negatively charged corneal surface [12].

##### Conjunctiva

1. Conjunctiva

The conjunctiva is a thin vascularized stratified cylindrical epithelium with its underlying stroma covering the exposed sclera and the eyelids. This membrane shows a 17-times higher surface area than the cornea [104], although conjunctiva is less permeable for lipophilic molecules. Hydrophilic compounds absorption is also reduced because of tight junctions in the corneal epithelium. Despite this fact, the conjunctival absorption is higher than the corneal’s due to the wider intercellular spacing allowing the passage of molecules up to 10 kDa. Furthermore, through the corneal route, via the conjunctiva, drugs are removed into the systemic circulation because of the presence of blood and lymphatic capillaries in the conjunctiva [12].

2. Blood–Aqueous Barrier (BAB)

**Figure 6:**
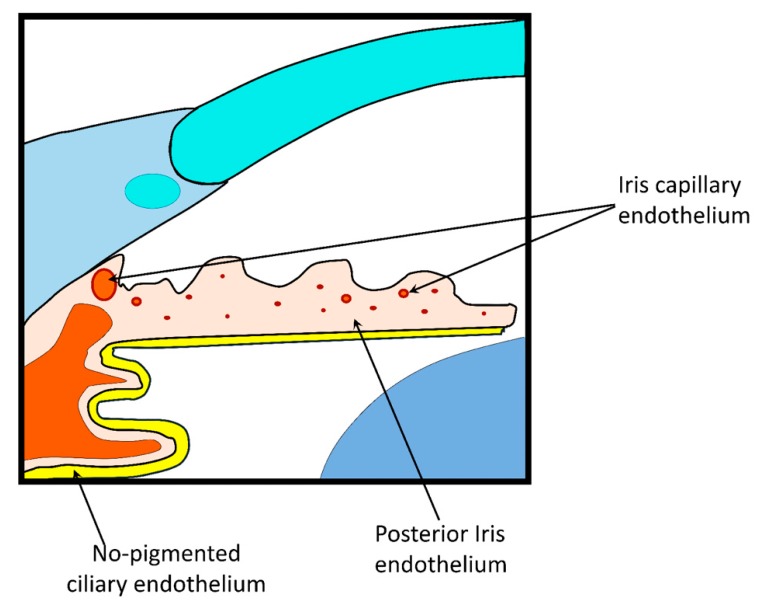
Blood–aqueous barrier (BAB) structure.

The BAB is formed by the iris and ciliary muscle vasculature endothelium and by the posterior iris and non-pigmented ciliary epithelium (Figure 6). This barrier is poorly permeable due to the tight intercellular junctions, which limit the passage of substances with high molecular weight or high hydrophilicity. This biological barrier is also responsible for the selective diffusion of ions and small solutes through space between cells. There are active transporters that alters drug permeation, depending their activity on the passive diffusion rate. However, their significance in drug absorption is still unclear and requires elucidation.

For systemically delivered drug, the tight junctions of the iris vasculature limit the passage of substances from the plasma to the iris stroma. The non-pigmented ciliary epithelium tight junctions restrict the passage of substances from ciliary body stroma to the posterior chamber [12,105]. Overall, this barrier modulates the passage of molecules between the anterior and posterior segment, as well as from plasma to aqueous humor. The drainage of aqueous humor (2.0 to 3.0 mL/min) also hinders the passage of drugs [106].

#### 2.2.3. Advantages and Limitations

Topical administration is the most challenging administration route for drugs intended to treat pathologies that are affecting the posterior segment of the eye. Once administered onto the ocular external surface, the drug absorption is restricted by the different barriers, these being: Static (cornea and the BAB), dynamic (conjunctival and lymphatic conjunctival lymphatic and lacrimal drainage), and metabolic (carriers and receptors) barriers [99]. Nevertheless, it is considered the least invasive method of ophthalmic application due to patient compliance and ease of administration. Moreover, it is not required the ophthalmologist intervention since eye drops can be instilled by the patients themselves [5]. However, significant disadvantages have been found using this administration route to the posterior segment, such as the significantly low bioavailability (1–5%) and ocular penetration (less than 0.001%) in the intraocular tissues (aqueous humor among others) [107]. The main reasons are that the drug applied topically is quickly removed by ocular protective mechanisms such as nasolacrimal drainage, blinking (6 to 15 times/minute), tear renewal (0.5–2.2 μL/min) and high lacrimal clearance [108,109]. Besides, the unproductive absorption to the systemic circulation through the conjunctiva, choroid, uveal tract and internal retina also limits the drug access to the posterior segment. Significant efforts towards the development of new drug delivery systems to the posterior segment of the eye have been carried out to overcome the obvious limitations of topical ocular administration.

#### 2.2.4. Pharmacokinetics

**Figure 7:**
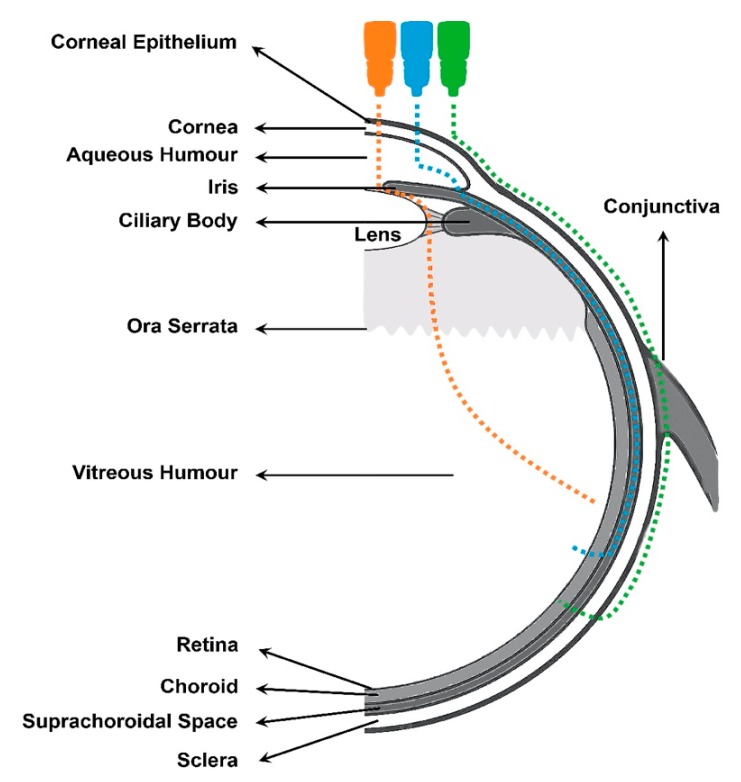
Different topical routes of drug absorption from the cornea/conjunctiva to the vitreous humor: Periocular route marked in green. Uvea-scleral route in blue. Transvitreal route marked in orange.

Drug topical absorption into the posterior segment of the eye strictly depends on the route through which the drug is distributed (Figure 7). Thus, the drug corneal exposure will allow for its concentration to be higher in the aqueous humor, whereas higher drug concentrations in the back of the eye will be reached after direct exposure on the conjunctival surface.

For example, the drug loss in the precorneal segment is because of the tear fluid. The administered drug will be released from the vehicle to the tear film, where tear fluid dynamics accelerate its elimination due to the complete replacement of the tear film every 2–3 min [99]. Furthermore, different enzymes and lacrimal proteins can influence the drug metabolism in the precorneal surface [107].

When administered topically, the low amount of drug that has been able to be absorbed will permeate from the cornea/conjunctiva surface to the retina across three different routes [98]: transvitreal route, uvea–scleral route and periocular route, being all of them conditioned by the nature of the drug, the pharmaceutical form and the anatomical characteristics of the eye.

The transvitreal pathway of absorption is the one followed by the drug via the transcorneal diffusion until it reaches the vitreous humor. The passage of molecules through the cornea depends on their lipophilicity, molecular weight, charge and ionization degree [110,111]. Once the drug is applied to the ocular surface by eye drops, the passage to the anterior chamber is driven by a diffusion process passing through the corneal epithelium via the paracellular or transcellular route. The penetration through these routes always depends on the drug concentration in the tear film and the drug retention time on the ocular surface. Hydrophilic drugs cross the corneal epithelium via the paracellular route while lipophilic drugs cross the epithelial barrier along the transcellular route. Once the drug crosses the cornea and reaches the aqueous humor, it is distributed to the surrounding tissues (ciliary, iris, crystalline body) and to the vitreous humor by diffusion process [98].

The aqueous humor is mainly drained (75–85%) through the Schlemm’s canal (tubular conduct with thin and porous walls that allows the absorption of large protein molecules). The rest of the elimination process is carried out through the iris and the connective tissue of the ciliary and suprachoroidal muscles, leading to the uvea towards the extraocular connective tissue. The drug removed from aqueous humor contributes to a decrease in the amount of drug that could be able to reach the posterior segment of the eye. 

The iris is one of the two ocular tissues that contain melanin, a pigment responsible for its color. Melanin can interact with some drugs, causing its retention in the iris. This retentive effect is more pronounced in case of lipophilic drugs, resulting in a drug sustained action [112] but decreases its pharmacological activity [5]. Overall, the drug bioavailability in the aqueous humor through this route has been determined to be less than 5% of the applied dose [113].

The uveal-scleral route followed by the administered drug after topical administration corresponds to the transcorneal diffusion to the anterior chamber and its drainage through the aqueous humor to the uvea–scleral tissue towards the posterior tissues.

This route follows the same absorption process through the cornea until reaching the aqueous humor as the transvitreal one. Once in the aqueous humor, the drug diffuses through the suprachoroidal space to the choroid causing an unproductive absorption. To a large extent, this is due to the drug partial elimination to the systemic circulation through the BAB capillary fenestrations, giving rise to the appearance of side effects. The drug is transported towards the systemic circulation through the choriocapillaris, which can cause side effects systemically. However, some other external factors, such as prostaglandins (drug penetration improvement to the back of the eye when they are co-administered via the uveo–scleral route) can affect the process [114,115]. The periocular drug absorption comprises the drug diffusion through the conjunctiva to the Tenon’s capsule and its diffusion through the sclera, choroid, and retina.

It also must be taken into account that between 34% and 79% of the administered dose on the ocular surface is primarily removed by this way into the systemic circulation due to the high degree of conjunctival vascularization and its large surface area (16–18 cm^2^) [113]. Therefore, for a drug to penetrate must have a molecular weight no greater than 70 kDa [116]. In addition, it should be considered that the drug transport through the conjunctiva, sclera, choroids and retina can occur by passive (paracellular or transcellular) or active routes (membrane carriers) [114,117]. Some lipophilic drugs can passively penetrate throughout the RPE by directly accessing the posterior segment through lateral scleral diffusion followed by penetration through the Bruch’s membrane and the RPE.

#### 2.2.5. Drug Delivery Systems

Currently, there are few commercial topical formulations specifically intended for the treatment of ocular posterior segment diseases. Even so, in recent years, new advanced topically drug release systems have been developed in order to improve the drug access to the back of the eye, such as nanoparticles [99,118], emulsions [119], nanostructured lipid carriers [120], liposomes [121], and nanosuspensions [122].

Despite the enormous efforts to develop efficient topical formulations intended to reach therapeutic drug concentrations in the posterior segment of the eye, the most relevant results have been obtained by using eye drops. In an ex vivo assays using cow eyes, the topical application of a memantine hydrochloride ring that functioned as a reservoir on the corneal surface demonstrated the efficient drug distribution of the into the choroid and retina [123]. Currently, memantine is in phase III studies in humans as a neuroprotector and neuroreparative drug in ophthalmic diseases. Another approach has been made by investigating the topical application of a brimonidine ocular solution in monkeys, rats, and rabbits. Results have shown that the formulation administered repeatedly reached an enough drug concentration to provide neuroprotection in the retina [124]. In the AMD treatment, promising results have been obtained in rabbit retina by the dexamethasone-cyclodextrin complexes topical application [125,126].

### 2.3. Systemic Administration

Systemic administration route constitutes an alternative route in the treatment of eye’s posterior segment pathologies. It is normally used as a coadjutant treatment or as a second choice after the failure of intravitreal or subconjunctival injections treatment. It is based on drug administration by conventional routes (e.g., oral, intravenous, or intramuscular). Once the drug reaches the bloodstream, the absorption will take place through conjunctival, episcleral and/or choroidal vessels to get the vitreous cavity, although most of the drugs do not pass the main blood–ocular barriers [6].

#### 2.3.1. Advantages and Limitations

Systemic drug administration shows a series of advantages and disadvantages compared to other administration routes to treat posterior segment diseases. Firstly, systemic administration is very effective in the case of concomitant ocular and systemic diseases as they can both be treated with only one treatment. Although ocular effects slowly appear with the systemic administration compared to other routes, the duration of the effect is more prolonged [127].

The oral administration to treat ocular diseases presents some positive features: Non-invasive, no need to use strict sterile conditions, high patient compliance, and adherence to treatment and width availability of pharmaceutical forms that provide great drug stability [5,103,128]. Oral administration is usually combined with topical ocular administration [5].

However, the systemic route presents certain drawbacks [5,103]. The presence of physiological barriers (BAB and BRB mainly) prevents the drug passage to the eye, leading to a drug bioavailability of less than 2% [5]. This low bioavailability forces the administration of high doses of the drug to obtain therapeutic concentrations into the vitreous which may lead to systemic toxicity and severe side effects. In addition, a lag time occurs between the drug administration and onset of pharmacological effect. An important prerequisite for a drug to be administered by oral route (for ocular applications) is to have a high drug oral bioavailability [5]. Even though, the limited access to the posterior segment of the eye implies the administration of high doses of drug or an increased administration frequency to obtain a significant therapeutic response [103,116,129]. However, these procedures can exacerbate drug toxicity as a consequence of the drug non-specific absorption to other organs [116,130,131]. Trained personnel are also required in case of intravenous or intramuscular administration.

Likewise, parenteral administration is also a systemic administration route used as an alternative pathway in posterior ocular pathologies. Ocular drugs can be administered by intravenous injection, although its use is less frequent than oral administration route [132].

#### 2.3.2. Pharmacokinetics

##### Absorption to Ocular Tissues

Systemically administered drugs can easily reach the choroid due to the high vascularization of this tissue, as more than 85% of the ocular blood flow takes place in this layer, with a value of 43 mL/h [133], and choriocapillaris fenestrations [5,130].

Drug transport from blood circulation to the ocular cavity is strictly regulated by two anatomical and physiological barriers: The BAB and the BRB [8,134]. These barriers limit drug bioavailability by restricting its intercellular permeation to the anterior and posterior segment of the eye. This barrier effect is mainly due to the presence of highly complex tight junctions among epithelial and endothelial cells, which are located on the interface between blood flow and intraocular tissue. Moreover, it was observed that the drug passage improves during inflammatory conditions [135]. This is due to an increase in vascular permeability that leads to greater extravasation of components from the bloodstream to extravascular tissue [136].

Drug entry into posterior ocular tissues is mainly governed by the BRB. Thus, the relationship between drug permeability and physicochemical factors has been demonstrated, concluding that drug permeability increases with decreasing molecular weight and/or protein binding but improves with increasing lipophilicity [127]. As a result, small and lipophilic compounds can easily cross eye barriers (RPE, non-pigmented internal ciliary epithelium and retinal blood vessels), while hydrophilic and large compounds penetration is restricted [133]. However, hydrophobicity and high molecular weight tend to increase drug’s half-life time in the posterior segment of the eye [137]. Therefore, RPE presents a selective permeability to highly hydrophobic drugs, whereas the penetration of hydrophilic and/or large substances being much more limited.

The type (influx or output flow) and/or location (vitreous or blood side) of the ocular transporters also condition the drug absorption and its concentration in the intraocular cavity [138].

Recently, many drug efflux pumps were identified in the ocular barriers. Among transporters with the greatest influence on the drug´s arrival to the posterior segment of the eye are the efflux transporters, these being a part of the ATP-binding cassette (ABC) protein family located in the RPE [139]. Specifically, two efflux pumps related to the development of chemoresistance were described, these being: P-glycoprotein (ABCB1) and the multidrug-resistance associated protein (MRP1) (ABCC1).

P-glycoprotein is an efflux protein located in the iris, cornea and ciliary muscle, as well as in conjunctival epithelium, non-pigmented ciliary epithelium and RPE. It is a 170 KDa membrane protein that is expressed on the apical surface of the aforementioned cells [139]. It actively promotes drug molecules’ exit from retinal endothelial cells, reducing drugs accumulation within them [139]. 

For its part, MRP1 is a 190 KDa efflux protein encoded by the MRP1 gene on chromosome 16p13.1 and bounded to the choroidal side of the retinal barrier. It is an atypical ABC transporter with two cytosolic nucleotide-binding domains (NBD) and seventeen membrane-spanning domains (MSD) [140]. It functions as a multispecific organic anion transporter, mediating an ATP-dependent transport of drugs and other xenobiotics [141]. In any case, molecular mechanisms of these drug transporters are not completely known.

##### Distribution to Ocular Tissues

In general, systemically administered drugs reach the target tissues from the blood. Drug plasma transport is protein-binding dependent, giving rise to (1) free drug fraction, which is active and susceptible of excretion/metabolism and (2) protein-bound fraction that acts as a drug inactive reservoir. Only the free drug fraction can cross biological membranes and, consequently, reach the ocular tissues.

Specifically, drug distribution through blood–ocular barriers is a key factor in the achievement of an effective ocular treatment with systemically administered drugs. Before mentioned ocular barriers regulate drug transfer between blood circulation and the eye in both directions. Drugs whose transport is predominantly carried out by passive diffusion, distribution and clearance are independent of the drug transport direction, although mediated permeation by transporters could lead to a drug preferred transport direction (inward or outward) [3,128].

In any case, it must be taken into account that, although the eye barriers structure and main permeability trends have been known for some time, the distribution process from the blood circulation to the posterior segment of the eye is not yet fully elucidated. However, pharmacokinetic simulation models have been created for the drug concentrations prediction in the vitreous humor, depending on the free drug concentration and the blood flow between the general circulation and the posterior segment of the eye [133].

Once drugs reach the vitreous humor, their distribution and elimination follow the same pattern as the followed after an intravitreal administration. The parameters that affect the drug pharmacokinetics within vitreous humor have been discussed in the section of intravitreal administration.

1. Proteins and Biological Binding

The free drug may accumulate more than expected in any posterior ocular layers if it binds to cellular components or if it acts as a substrate for a significant active transport process. Specifically, drugs binding to ocular tissues’ proteins and pigments, mainly melanin, significantly affects their transport to the posterior segment of the eye [6,130,142,143].

Melanin is a polyanionic biological pigment located in the uvea and the RPE in the ocular tissues as melanosomes or pigment granules, which are melanoprotein complexes where melanin is bound to a protein matrix [142]. Menon et al. determined the existence of 6–8 mg melanin at the ocular level [6,144].

Drug binding to melanin and proteins in ocular tissues is an important factor in drugs administration by systemic route since they can modify the drug bioavailability in the target site and, consequently, reduce its pharmacological activity [5,145]. 

Specifically, ocular melanin has a significant influence on drug pharmacokinetics and permeation through the retina [5,146] due to its capacity to bind (mainly reversible binding) free radicals and chemicals, especially basic (pKa values above 7) and lipophilic drugs by electrostatic, charge transfer, and van der Waals forces [5,147]. Main pharmacological consequences of the drug-melanin complexes are drug accumulation and retinal toxicity, besides the fact that larger doses are needed to obtain a therapeutic response due to the bound-to-melanin drug inactivation (e.g., betaxolol, metoprolol, and phosphodiesther oligonucleotides) [142]. Nevertheless, this drug depot may act as a reservoir over a long time, prolonging drug effects [145,146].

##### Drug Elimination

Regarding drug elimination process by this route, it must be taken into account that the administered drug not only faces passive barriers but also active barriers, such as clearance through the choroidal blood flow, which is extremely high, and the number and size of the choriocapillaris fenestrations that make up this system [6,148], by presenting a 70–80 nm pore size and a number between 30–50 fenestrations/μm^2^ [149,150].

In general, drug elimination from the posterior segment of the eye can be carried out by two different ways, anterior and/or posterior pathways [103], following the same pattern described for the intravitreal administration route.

#### 2.3.3. Drug Delivery Systems

The design of ophthalmic drugs systemic administration forms is aimed at achieving therapeutic concentrations in the posterior segment of the eye without causing undesired side effects. Drug targeted administration to these tissues from the systemic circulation has only been studied in preclinical animal models. Studies were carried out based on qualitative investigations performance and assessed by microscopy, immunohistochemistry, and/or angiography techniques [3]. Some of the novel systemic targeting systems studied for drug transport through the choroid to the posterior segment of the eye include [137]: 20 nm gold nanoparticles [151], polyethylene glycol (PEG) conjugated immunoliposomes [103], PLGA nanoparticles or Visudyne^®^. Currently, none of them is commercialized except for Visudyne^®^, an intravenous administration formulation used in photodynamic therapy for age-related wet macular degradation treatment [151].

Even so, several strategies related to the design of advanced delivery systems are currently under study, such as: (1) Structurally modified drugs that can effectively avoid MRP1 efflux transporter to improve ocular penetration, (2) hydrophilic drug administration through advanced delivery systems directed by transporter/receiver (superficially marked systems with an ocular-receptor specific substrate and/or vehicles attached to substrates that show a high affinity for ocular tissues) [139], and (3) drug affinity improvement to BRB transporters, such as amino acid, oligopeptides or cation and anion transporters, in order to increase drug transport to the posterior segment of the eye [152].

#### 2.3.4. Systemic Drugs for Posterior Segment Eye Diseases

Drug systemic administration is not the preferable route in the treatment of posterior ocular segment pathologies, although it is still useful in many cases, being an administration pathway carried out by different routes (e.g., oral, intravenous, intramuscular). Thus, some drugs are administered orally (see Table 4).

Apart from these, orally administration formulations with antineoplastic and antiviral agents have also been studied [103]. In relation to the latter one, it must be considered that the most frequent ocular posterior segment pathologies with viral etiology are associated with immunosuppression states (e.g., AIDS or transplants). These include: Cytomegalovirus retinitis, whose treatment is based on the antivirals intravenous administration (valganciclovir, ganciclovir, foscarnet, and/or cidofovir) as well as acute retinal necrosis and progressive external retinal necrosis, whose etiology is broad (varicella-zoster, herpes simplex, cytomegalovirus, or Epstein-Bar virus) and whose treatment is based on the acyclovir (very effective), ganciclovir or foscarnet administration [159].

Similarly, parenteral administration formulations have also been developed for the same purpose, including different intramuscular and intravenous preparations. These encompass: Parenteral antibodies for the uveitis treatment [3] and hydroxocobalamin intramuscular injections (B12 vitamin, Alpha Redisol) for the treatment of B12 vitamin deficiency states, as well as certain antibiotics (e.g., penicillin, gentamicin, ceftazidime, or amikacin) used in the subsequent ocular infections treatment (uveitis, scleritis, and/or pseudoscleritis) (see Table 5).

Likewise, the antibiotic combinations administration by continuous perfusion for the serious-eye-diseases treatment, such as endophthalmitis, is quite frequent [127,160]. As an example, ceftazidime is the best studied cephalosporine due to its activity spectrum against Gram-negative bacilli (including *Pseudomonas aeruginosa*). It is more frequently used as a first election treatment in this pathology, in monotherapy or combined with other antibiotics, since it allows reaching ocular concentrations in the order of 35.4 mg/l after intravenous administration of a 100 mg/kg antibiotic dose [161].

In addition to these, different intravenous drug delivery strategies have been tested for drug arrival to the posterior segment of the eye [168]. Specifically, photodynamic therapy with verteporfin has been practiced, being a choroidal neovascularization effective treatment by stopping the neovascular membrane growth. Apart from this, ocular posterior segment diagnostic intravenous techniques have also been developed, being the most prominent the fluorescein digital angiography, an exploratory technique for the retinal vasculature visualization by means of the sodium fluorescein injection (vegetable origin orange dye).

In order to point out, eyes can be also exposed to systemic drugs (as a kind of side effects) and xenobiotics not intended for ophthalmic treatment, such as bisphosphonates (whose action mechanism is based on the bone-resorption inhibition, being used in the osteoporosis prevention and treatment), which can cause ocular inflammatory reactions (uveitis, neuritis, iritis, scleritis, or pseudoscleritis) [169]. Reformulation studies have also been carried out on these drugs in order to reduce their passage from the blood to the eye, making them more selective in order to decrease associated ocular side effects [169].

### 2.4. Periocular Administration

The periocular route has been considered as a promising drug administration route for the posterior ocular segment diseases treatment due to the high concentrations obtained with this kind of formulations’ inoculation. This route allows the drug deposition on the scleral external surface and includes the following administration routes: subconjunctival, subTenon´s, retrobulbar, peribulbar and posterior juxtascleral. These pathways differ from one another in the location and/or injection direction in the proximity of the sclera.

#### 2.4.1. Advantages and Limitations

Periocular injections are associated with greater adherence to treatment by patients compared to intravitreal injections [139] since it is considered a less invasive administration route and capable of providing a relatively great drug bioavailability in the posterior ocular segment [150,170]. 

The sclera provides a relatively large area for the drug absorption (approximately 17 cm^2^) [171] compared to other ocular surfaces, like the cornea. Moreover, periocular administration takes advantage of the high scleral permeability. These two factors contribute to the potential effectiveness of the periocular administration compared to other ocular routes. 

On the other hand, the main drawback is that the drug needs to diffuse from the site injection to the target site, with the possibility of drug losses.

#### 2.4.2. Pharmacokinetics

In general, although this type of administration avoids the corneal-conjunctive barrier, the drug must cross several barriers to reach the target sites in the choroid, the RPE, or the neural retina [65,150]. To do this, it must overcome several dynamic, static, and metabolic barriers that limit drug access to the posterior segment of the eye. Two different types of barriers should be mentioned: (1) Physical barriers, which include sclera, choroid (its high blood flow can remove a significant fraction of drug before it can reach neural retina), and RPE, and (2) physiological barriers, as occurs with conjunctival, episcleral, and choroidal lymphatic flow [150], whose drug elimination ability is relatively fast [3,65,103]. In any case, episcleral blood and lymphatic flows are seen as the main limiting factors in drug periocular distribution, while anatomical barriers and choroidal blood flow are less important [116,150,170].

As a result, drug is removed into the systemic circulation, decreasing ocular bioavailability thereof. However, molecules that escape from the conjunctival vasculature can penetrate through the sclera and choroid to reach the neural retina and photoreceptor cells. In addition, permeability through sclera is lipophilicity-independent (e.g., inulin, methazolamide, pilocarpine, hydrocortisone) [172], unlike corneal and conjunctival layers, being dependent on molecular radio [4,42,137,148].

Drug reflux after periocular administration is the initial loss factor and contributes to its low bioavailability in the posterior segment of the eye [116,173]. It was demonstrated that the use of an adequate injection technique, volume and/or formulation type can improve drug bioavailability and ocular penetration [116,173,174,175].

##### Absorption

Drugs administered by periocular injections can reach its target site in the posterior segment of the eye through three different routes: Transscleral or direct penetration route, systemic circulation route, and anterior route [6,176]. In the anterior route, the drug diffuses directly through the sclera and the ciliary body, or indirectly through the lacrimal fluid and the cornea because of the conjunctival circulation reflux. In the systemic circulation route, the drug goes to systemic circulation through conjunctival, episcleral and/or choroidal vessels, where it is diluted, and then it returns to the eye by the blood flow. In the direct penetration route, drug reaches the vitreous humor through the underlying tissues; it represents the most important absorption route in terms of drug penetration and distribution to the posterior chamber of the eye.

In any case, it should be taken into consideration that the scleral permeability depends on the molecular radio, scleral hydration, and intraocular pressure [176,177] instead of molecular lipophilia [17]. Although the latter improves permeability through the RPE, it also increases drug loss from the choroidal and subconjunctival space to the bloodstream [3]. Regardless of the absorption route, only a small portion of the drug reaches the posterior segment of the eye [116,178] mainly due to the drug loss through periocular space, BRB, choroidal circulation, efflux transporters, and drug binding to ocular tissue proteins [116].

##### Elimination

Once the drug reached its target site, small molecules are rapidly removed from the administration site, presumably through conjunctival and episcleral blood and lymphatic flow [103,139,150], whereas larger molecules have much slower elimination kinetics, around 10 times lower [150], so that their residence time is much greater.

#### 2.4.3. Subconjunctival Route

Subconjunctival injection has been used to administer drugs in the anterior segment of the eye, achieving higher concentrations in the anterior chamber compared to the topical administration. However, this route has also been investigated as an alternative pathway to intravitreal injection for the drug administration of retinal diseases treatment [6] due to the fact that it is considered a less invasive technique, comparing it with the intravitreal administration route [116,179]. It also minimizes the side effects, mainly endophthalmitis, cataracts, and retinal damage [116].

The drug is injected under the conjunctival membrane that covers the sclera, avoiding the conjunctival epithelial barrier. In this way, direct access to the transscleral route is achieved [116], increasing its bioavailability in aqueous humor in comparison with the topical route, which presents the corneal barrier as an impediment.

##### Advantages and Limitations

Subconjunctival administration shows two effective types of sustained drug delivery to the retina, both the topical and intravitreal administration. Moreover, it is an alternative route in order to allow easy and better accessibility and reduce the side effects caused by the intravitreal injection procedure (e.g., intraocular pressure and cataracts) [3]. 

The injection volume with the same drug concentration could be higher in this route in comparison with the intravitreal injection [3], enabling a wide dose range. In addition, the enzyme absence in the injection area is an important advantage due to the low enzymatic drug degradation.

The availability of pharmacokinetic and pharmacodynamic data about this route is limited. However, the drug delivery to the back of the eye through this route is better compared with topical and systemic administration routes [150].

This pathway also has some limitations regarding the concentration that can cross to the retina. The elimination via systemic circulation and to the tear cause a reduced bioavailability. Nevertheless, the permeability is modified by the age, according to the patient get older, the permeation through the sclera is less prevented so the amount of drug that can reach the retina is higher.

##### Pharmacokinetics

The permeation speed through the sclera is drug size-dependent [17], where the small molecules movement is constant. Macromolecules permeation is slower [3,150,172,180], although up to 70 kDa size molecules can easily penetrate the sclera [17,33,116,181,182,183]. Once the sclera is crossed, a rapid elimination caused by the choroidal blood flow takes place due to its large choriocapillaris fenestrations. Molecules that pass through this barrier faster and better are the small lipophilic ones compared to macromolecules [3,33,172,184]. However, small molecules are also quickly removed. 

Likewise, lymphatic flow plays an important role in the drug elimination since conjunctival lymphatic vessels represent 50% of the surface [185]. Once drug crosses the sclera, it must break through the choroid and the RPE to reach the vitreous humor. For all these reasons, between 80–95% of small molecules drain into the systemic circulation [150]. Approximately, 10% of the drug is available for the anterior segment of the eye and only about 0.1% reaches the retina [3]. Thus, high initial drug concentrations are needed in order to achieve effective levels in the posterior segment due to the deficient bioavailability observed.

The injection volume is an important factor in the subconjunctivally administered drug pharmacokinetics as the drug reflux from the injection site is the initial loss factor that contributes to its low bioavailability [116,173]. Adequate volumes should be used in order to achieve high drug levels in the back of the eye. If large volumes are administered (over 200 μL), the reflux of drug solution out of the injection site increases and a greater drug permeation to the aqueous humor is obtained.

##### Drug Delivery Systems

Different techniques have been developed in order to increase the subconjunctival administered drug penetration through the sclera. One of these is the transscleral iontophoresis, an electrodynamic technique that may improve the periocular injections efficacy in some ophthalmic drugs (see Table 6), such as corticosteroids, antibiotics, NSAIDs, and immunosuppressants [186,187].

Nano- and microparticles are interesting sustained drug delivery systems for treating posterior segment diseases. These types of systems were tested, obtaining promising results for the VEGF inhibition with corticosteroids (budesonide and dexamethasone) and selective COX2 inhibitors (celecoxib) [188,189,190,191,192].

Thermo-responsive hydrogels are also systems that can be used to facilitate a sustained drug delivery. This pharmaceutical form turns into a gel when contacting with the injection area due to a temperature difference. These systems are employed as an alternative to the classic intravitreal route mainly because of its minimally invasive procedure. Subconjunctival placement of insulin-impregnated hydrogels has presented advantages over topical and intravitreal injection in DR treatment [197,198].

#### 2.4.4. SubTenon’s Route

SubTenon’s route is one of the most promising routes to reach the eye posterior segment due to the possibility of obtaining better pharmacokinetic profiles of the administered drugs [116,130,199]. SubTenon’s injection is placed in an avascular area between Tenon´s capsule and sclera, around the upper portion of the eye and into the belly of the superior rectus muscle [179,200]. It is divided into anterior and posterior segments at the insertions of extraocular muscles and their associated fasciae [179,201]. 

##### Advantages and Limitations

As a general idea, subconjunctival injections show a better drug efficacy in the anterior segment, while subTenon’s injections lead to an increased penetration to the posterior segment of the eye [202,203]. 

Drug passage to systemic circulation is reduced and the contact time with the sclera is prolonged as a consequence of the pharmaceutical form injection. Nevertheless, drug elimination caused by the choroidal circulation can produce a shortened duration of action [130]. 

Besides that, it should bear in mind that Tenon´s capsule degeneration is age-related, which leads to an easier drug diffusion towards the retrobulbar cone. As a result, better drug levels are achieved in the posterior segment of the eye [130].

##### Pharmacokinetics

A 2.5 cm long blunt-tipped cannula needle is usually employed in the injection procedure into the Tenon´s capsule after a surgical incision. This is a widely used technique for local anesthesia during ocular surgery because the cannula approach avoids sharp-needle complications [103,179]. The formulation volume is up to 4 mL and is injected around the muscle belly, behind the equator [179]. The possibility to inject a large volume into the subTenon’s space causes a lower and slower clearance and a more readily transscleral drug delivery [199].

Some of the most employed applications by using subTenon’s administration route are: corticosteroid injections (triamcinolone acetonide (TA)) for chronic posterior uveitis [204] and cystoid macular edema treatment (see Table 7) [205].

Comparing the intravitreal effects versus subTenon’s TA administration for diabetic macular edema (DMA), results showed that intravitreal TA injection had greater clinical effects than the subTenon’s injection [206,207]. Nevertheless, a lower incidence of serious complications has been shown with subTenon’s injections than the intravitreal ones [206,208].

#### 2.4.5. Retrobulbar Route

Retrobulbar administration arises as an alternative route to the drug targeting the posterior ocular pathologies treatment where the optic nerve or retrobulbar spaces are involved. Injection procedure is based on drug inoculation into the space between the orbital rim and the balloon edge [176], the retrobulbar space, which lies inside the extraocular muscle cone, behind the globe, being a deeper injection and uses less volume (comparing it with the peribulbar one). The usual procedure for the retrobulbar injection implies a 3 mm insertion beyond the posterior surface of the eyeball and the maximum volumes of injections are 3–4 mL.

##### Advantages and Limitations

Complications associated with retrobulbar injections, although potentially severe, are uncommon. Possible complications of retrobulbar injection include retrobulbar hemorrhage, globe perforation, optic nerve damage, respiratory arrest, optic atrophy, retinal vascular occlusion, and extraocular muscle myopathy [210]. This route has serious risks of causing ocular damage due to the proximity of the optic, motor and sensory nerves [151].

As retrobulbar injections are performed outside the ocular globe, there is little or no influence on intraocular pressure, in contrast to the intravitreal injections [179]. Compared to peribulbar injections, retrobulbar administration is a deeper injection and uses less volume. 

Moreover, an injection up to 5 mL may be made, which is a considerably higher volume compared to the other types of administration to treat the ocular posterior segment diseases. This means that higher amounts of drug can be administered. 

Retrobulbar administration is mainly used for corticosteroids administration as an alternative treatment to reduce the posterior segment inflammation. It is also used for local anesthesia injection in certain intraocular surgeries, where drugs are directly administered into the muscular cone, an area located among four eye muscles in the back of the globe.

#### 2.4.6. Peribulbar Route

Peribulbar administration enables drug injection into the muscular extraconal compartment of the eye, also called peribulbar space, which is externally localized to the rectus muscles.

##### Advantages and Limitations

It is frequently preferred for its ocular complications’ low rate, theoretical safety and ease of application comparing it with the retrobulbar route. Although both routes showed to be useful in anesthesia or akinesia, their use is limited due to the procedure associated complications (e.g., eyeball perforation, oculomotor reflex stimulation, optic nerve trauma, and orbital hemorrhage) [160]. Moreover, the peribulbar administration is less effective for anesthetizing the eye than the retrobulbar route, although a higher volume, up to 8–10 mL, may be injected [181]. However, this technique is slower and less efficient than retrobulbar injection, besides the fact that it requires multiple injections and larger volume of the injected formulation. It also carries a higher risk of potential chemosis and an intraocular pressure increase [211]. 

Currently, the peribulbar approach is a widely used method for the local anesthesia administration. The injection procedure has been briefly described for the administration of lidocaine and bupivacaine mixture as a local anesthesia pre-treatment in modern intraocular surgery [160]. A mixture of peribulbar bupivacaine combined with TA and cefazolin was also studied as a prophylactic treatment to reduce pain and possible inflammation and/or infection associated with vitreoretinal surgery [212].

#### 2.4.7. Posterior Juxtascleral Route

The posterior juxtascleral injection, recently developed by Alcon Laboratories^®^, is an alternative administration route based on the therapeutic agent deposition directly in the closest area to the macula, without puncturing the eyeball [116]. It allows achieving higher drug concentrations in the target site due to the fact that the scleral thickness decreases near the equatorial region, an aspect that promotes a greater drug penetration towards the posterior segment of the eye [3,65]. 

This novel technique requires inserting a blunt-curved cannula after a conjunctival incision. The curved cannula follows the scleral surface without puncturing the ocular globe and drug is injected into the juxtascleral space overlying the macula. Besides, no clinically significant safety and efficacy issues related to this administration pathway were reported [213].

In addition, this route reaches a drug therapeutic dose for up to 6 months in the macular region [174], avoiding the risk of intraocular damage and reducing side effects, such as endophthalmitis, retinal detachment and glaucoma, among others [116,174]. Besides, drug reflux, which occurs when the drug flows back out of the incision made at the insertion site, may affect drug efficacy, although Alcon Laboratories^®^ have developed a counter pressure device to deal with this side effect. There is an increased risk of systemic drug exposure due to the drug contact with the orbital tissue when compared with intravitreal injections, though there is much less systemic exposure than occurs with either topical or systemic therapy. 

Currently, there are few commercial formulations which are administrated by using this administration route, such as anecortave acetate (Retaane^®^) [214], a cortisol synthetic analogue used for AMD treatment and prevention, or TA for DMA treatment unresponsive to laser photocoagulation [213].

#### 2.4.8. Suprachoroidal Route

Suprachoroidal injection was introduced as a potential drug administration route to the posterior segment of the eye although it is not clinically used at present [150]. Drug is injected into the suprachoroidal space below the sclera inner surface using microneedles (micron-dimensions needles that can infuse fluid into tissue with excellent spatial targeting), up to 50 µl maximum volume. Formulation is distributed throughout the suprachoroidal space as a consequence of the pressure exerted by the injection process itself [215].

The suprachoroidal space circumferentially goes around the eye and is located between the sclera and choroid, leading to reach the macular area [216,217]. This administration route was tested for drug delivery into the posterior segment of the eye employing surgical procedures (by introducing catheters into this space) and gave rise to a long-term therapy, where drugs could be targeted towards the choroid and retina by direct contact with the administration site [218,219].

##### Advantages and Limitations

Suprachoroidal delivery route theoretically presents several key advantages compared to the standard intravitreal injections [220], such as that this space does not interfere with the optical pathways and it is a good pathway to target diseases at the outer retina, photoreceptors, RPE and choroid. Moreover, its diffusional pathways and pharmacokinetics are completely different from the intravitreal one, being a possible resourceful way to target drugs to the posterior segment of the eye. In comparison to other periocular administration routes, drug diffusion from the suprachoroidal space avoids several ocular barriers (e.g., cornea, conjunctiva and sclera). Additionally, the suprachoroidal space could act as a potential reservoir inside the eye and sustained release formulations or devices could optimize diffusional kinetics from this space. Finally, this space provides a safer way with larger biologic and/or immunogenic agents [220]. There are also disadvantages involved, such as suprachoroidal hemorrhage and choroidal detachment [221].

However, the access to the suprachoroidal space is complicated and the methods used were usually invasive and too complex to be performed as a simple office procedure [218,219]. Pre- and post-injection histology demonstrated that the potential space of the suprachoroidal region returns to a normal configuration after a brief time period [220]. Also, using die-casting methods, the suprachoroidal space is rather extensive and has the capacity to expand and accommodate a relatively large volume of material [220]. Delivery device parameters’ optimization showed that microneedle length, pressure and particle size played an important role in determining a successful delivery into the suprachoroidal space. 800–1000 μm needle lengths, and 250–300 kPa applied pressures provided the most reliable delivery [215]. The usual injection volume for the suprachoroidal administration has been determined to be 25 µl [220].

##### Pharmacokinetics

Suprachoroidal administration leads to greater vitreous bioavailability for small lipophilic (1.5%) and hydrophilic (0.19%) molecules, while macromolecules (4.2%) show a 6–23 times improvement in terms of ocular bioavailability compared to the drug administration by subconjunctival route [150].

Compared with small molecules, macromolecules have a much longer half-life time in ocular tissues where steady state is slowly reached. Drug levels in the vitreous humor are generally reached 15 h for small lipophilic drugs, 70 h later for small hydrophilic drugs and 500 h later for larger molecules. Therefore, it was seen steady-state drug levels are quickly reached for small molecules, becoming 100–1000 times slower for macromolecules [150].

Several studies showed a significant fraction of the drug administered by the suprachoroidal route was removed through conjunctival and regional lymphatic nodes, including those of high molecular weight [6,175]. In addition, it should be taken into account that choroidal blood flow also removes a huge part of the inoculated drug (96–99%) in the case of low molecular weight compounds, whereas high molecular weight drugs will be removed from the tissue almost equally through the choriocapillaris (54%) and the subconjunctival space (41%) [150].

##### Drug Delivery Systems

Currently, there are no pharmaceutical forms commercialized to be administered by suprachoroidal injections. Nevertheless, this type of administration was widely studied by using fluorescein and fluorescently tagged dextrans (40 and 250 kDa), bevacizumab and polymeric particles (20 nm to 10 µm in diameter). Sulforhodamine B microneedle injection was also studied as well as nanoparticle and microparticle suspensions into the suprachoroidal space [215,222]. TA formulations were also researched as a way of DMA alternative treatment, as well as ranibizumab and bevacizumab injections, although recent studies showed these large biologic proteins are quickly removed from the suprachoroidal space [220].

#### 2.4.9. Subretinal Route

Drug subretinal route has emerged as an alternative administration route to intravitreal administration due to its side effects and lower adherence to treatment by patients derived from the latter [223,224]. Thus, subretinal administration involves drug inoculation into the subretinal space [224], an ocular space located between RPE cells and photoreceptors [224,225].

##### Advantages and Limitations

Subretinal injection is an especially useful route for the posterior ocular pathologies treatment by providing a direct route with a very precise location through a minimally invasive injection. A typical volume of around 150 µl is injected, leading to a transient detachment between these two layers [2]. A lower drug dose is needed to accurately reach subretinal-space cells.

Compared to intravitreal administration, subretinal injection has a direct effect on subretinal space cells. Unlike vitreous cavity, subretinal space is an isolated closed anatomical area, which also has a greater immunological defense mechanism providing a safer route of administration in case of entry of bacteria [178,224].

Basically, three subretinal injection techniques have been studied, these being: (1) A transcorneal route through the pupil, surrounding the lens, and passing the vitreous humor and the retina [226], (2) a transscleral route through pars plana or limbus, crossing the vitreous humor and the opposite side of the retina into the subretinal space [227], and (3) a transscleral route through the choroid and Bruch’s membrane without penetrating the retina [224]. All routes were equally effective regardless of the chosen one. The administration procedure is performed under direct visualization by using a surgical microscope and where blister formation should be observed as a sign of the procedure success.

##### Drug Delivery Systems

Subretinal administration has been considered as one of the best strategies for gene therapy using viral vectors, a carried-out treatment effectively achieved for pigmentary retinitis (PR) and Leber’s congenital amaurosis (LCA) [225,227]. Gene expression is however limited to the injection site, suggesting that the primary barrier for efficient therapy following subretinal injection is the retina itself [2]. In addition, it was reported macrophages subretinal injection leads to pathological fibrosis, which could be used for the advanced AMD evaluation [224]. Subretinal delivery can also be used for stem cell transplantation in ocular degenerative diseases, which was reported in vivo studies and aimed at clinical applications [224].

## 3. Conclusions

The eye is one of the most inaccessible organs in terms of obtaining therapeutic drug concentrations, especially in the treatment of posterior segment ocular pathologies. Conventional administration pathways, such as topical or systemic routes, usually show important limitations, either by low ocular penetration or by the appearance of side effects linked to the posology, among others.

New drug delivery systems (DDS) are needed to prolong the administration intervals for posterior segment ocular pathologies, even though the development of novel DDS is particularly complicated due to several aspects must be considered, such as pharmacokinetics, immunogenicity, biodegradation, tolerability and toxicity, among others. Apart from these, different technological requirements must be taken into account, including reproducible manufacturing, clinical performance and sterility.

In the last few decades, an exponential increase in the design and development of novel DDS intended for the treatment of posterior segment ocular pathologies was observed. Biodegradable and non-biodegradable implants, microparticles, nanoparticles, microneedles, and microelectromechanical systems are the most innovative ones. Unfortunately, knowledge about drug targeting to the posterior segment of the eye is still sparse and, thus, there is not many DDS in clinical trials. Even so, memantine ophthalmic formulations as neuroprotective are in III phase studies at the moment, inter alia.

Likewise, huge advances were made in terms of research and development of new alternative administration routes to the posterior segment of the eye, including intravitreal and periocular pathways as the most relevant ones, as well as their subtypes. All of them, including a conventional administration routes (topical and systemic) show a series of specific pharmacokinetic characteristics, making them useful for the treatment of certain posterior segment ocular pathologies. However, these pathways have shown several advantages and limitations, where the election of one or another is dependent on, not just the pathology itself, but the pharmaceutical form, the drug used and the patient´s adherence to treatment, among others.

## Figures and Tables

**Table 1:** Vitreous half-life times for intravitreally administered drugs with different pharmacokinetic characteristics.

Pharmacologic Group	Drug	Characteristics	Half-Life Time (h)	Ref.
Corticosteroids	Dexamethasone	Low molecular weight Water insoluble	3.48	[66]
Antibiotics	Ceftizoxime	Low molecular weight Water soluble	5.70	[67]
Somatostatin analogues	Octreotide acetate	High molecular weight Water soluble	16.00	[68]
Antiviral	ISIS 2922	High molecular weight Water soluble	62.00	[69]

**Table 2:** Comparison of the anterior and posterior route of drug elimination from the vitreous humor [65,70].

Features	Anterior Route	Posterior Route
Tissue involved	BAB	BRB
Elimination pathway	Aqueous humor outflow	Choroidal flow
Molecule characteristics	Hydrophilic High molecular weight	Lipophilic Small molecular weight

**Table 3:** Summary of the main key pharmacokinetic parameters for different intravitreally administered drugs.

Pharmacologic Group	Subgroup/Drug	Half-Life Time (h)	MRT (h)	C_max_ (µg/mL)	Ref.
Nonsteroidal anti-inflammatory drugs	Ketorolac	4.3	6.16	175	[83]
	Diclofenac	2.05	2.95	65	[83]
Antibiotics	Penicillines	10–20	5–25	1000–5000	[84,85,86]
	Cephalosporines	5–15	5–30	1000–2250	[48,85,87]
	Tetracyclines	10–20	NA	125–400	[86,88]
	Fluoroquinolones	3.5–5.5	0.25–5	100–500	[49,89,90]
	Monobactams	7.5	NA	1000	[91]
	Carbapenems	2.5–10	NA	50–100	[92,93]
	Macrolides	40–60	NA	100–200	[85,94,95]
Antibodies	Bevacizumab	4.32	5.92	400	[96]
	Ranibizumab	2.88	4.03	162	[97]

**Table 4:** Summary of orally administered drugs for the treatment of posterior segment ocular diseases.

Pharmacologic Group	Drug	Pathology	Administration Route	Reference
Analgesics	Paracetamol	Ocular trauma treatment-associated pain	Oral	[153]
	NSAIDs (Flurbiprofen, Ketorolac, Diclofenac, Bromfenac and Nepafenac)	Ocular trauma treatment-associated pain	Oral	[153]
Antibiotics	Doxycycline	Neovascularization	Oral	[110,154]
	Tetracycline	Ocular rosacea	Oral	[111,155]
	Erythromycin	Orbital cellulitis	Oral	[111]
	Minomycline	Ocular rosacea	Oral	[110]
Corticosteroids	Dexamethasone	Giant cell arteritis	Oral	[153]
Immunosuppressants	Cyclosporine	Idiopathy or related-to-Behçet’s-disease uveitis	Oral	[156]
Carbonic anhydrase inhibitors	Acetazolamide (Diamox sequel^®^)	Glaucoma	Oral	[113,157]
	Etoxolamide	Glaucoma	Oral	[114,158]

**Table 5:** Summary of systemically administered drugs for the treatment of posterior segment ocular diseases.

Pharmacologic Group	Drug	Pathology	Target	Route	Reference
Antibodies	Secukinumab Tocilizumab	Uveitis	Inflammatory cytokines	Intravenous	[162,163]
	Ustekinumab			Subcutaneous	
	Abatacept		T-cell activation		
	Rituximab		B-cell targeting	Subcutaneous	
Vitamins	B12	Vitamin B12 Deficiency Optic Neuropathy	Folate receptor	Intramuscular	[164]
Antibiotics	Penicillin Gentamicin Ceftazidime Amikacin	Uveitis Scleritis Pseudoscleritis Endophtalmitis	Bacteria	Intravenous	[165,166,167]

**Table 6:** Summary of subconjunctival administered drugs for the treatment of posterior segment ocular diseases.

Pharmacologic Group	Drug	Pathology	Reference
Antidiabetics	Insulin	Diabetic retinopathy	[193]
Chemotherapeutics	Carboplatin	Retinoblastoma	[194]
	Topotecan		
Folic acid analogues	Methotrexate	Granulomatous panuveitis	[195]
Corticosteroids	Dexamethasone	Uveitis	[196]

**Table 7:** Vitreous pharmacokinetic parameters for long-acting triamcinolone acetonide subTenon’s injection.

Pharmacologic Group	Drug	Half-Life Time (days)	MRT (days)	C_max_ (ng/mL)	T_max_ (h)	Reference
Corticosteroids	Triamcinolone acetonide	17.1	23.1	22	24	[209]

## References

[1] WHO Team (2019). World Report on Vision.

[2] Peynshaert K., Devoldere J., De Smedt S.C., Remaut K. (2018). In vitro and ex vivo models to study drug delivery barriers in the posterior segment of the eye. Adv. Drug Deliv. Rev..

[3] Del Amo E.M., Rimpelä A.-K., Heikkinen E., Kari O.K., Ramsay E., Lajunen T., Schmitt M., Pelkonen L., Bhattacharya M., Richardson D. (2017). Pharmacokinetic aspects of retinal drug delivery. Prog. Retin. Eye Res..

[4] Hosoya K., Tachikawa M. (2009). Inner Blood-Retinal Barrier Transporters: Role of Retinal Drug Delivery. Pharm. Res..

[5] Gaudana R., Ananthula H.K., Parenky A., Mitra A.K. (2010). Ocular drug delivery. AAPS J..

[6] Ranta V.-P., Urtti A. (2006). Transscleral drug delivery to the posterior eye: Prospects of pharmacokinetic modeling. Adv. Drug Deliv. Rev..

[7] Wilson C.G., Tan L.E., Mains J. (2011). Principles of Retinal Drug Delivery from Within the Vitreous. Drug Product Development for the Back of the Eye.

[8] Cunha-Vaz J.G. (2004). The blood–retinal barriers system. Basic concept sand clinical evaluation. Exp. Eye Res..

[9] Gunda S., Hariharan S., Mitra A.K. (2006). Corneal absorption and anterior chamber pharmacokinetics of dipeptide monoester prodrugs of ganciclovir (GCV): In vivo comparative evaluation of these prodrugs with Val-GCV and GCV in rabbits. J. Ocul. Pharmacol. Ther. Off. J. Assoc. Ocul. Pharmacol. Ther..

[10] Zambito Y., Zaino C., Di Colo G. (2006). Effects of N-trimethylchitosan on transcellular and paracellular transcorneal drug transport. Eur. J. Pharm. Biopharm..

[11] Dey S., Mitra A.K. (2005). Transporters and receptors in ocular drug delivery: Opportunities and challenges. Expert Opin. Drug Deliv..

[12] Huang D., Chen Y.-S., Rupenthal I.D. (2018). Overcoming ocular drug delivery barriers through the use of physical forces. Adv. Drug Deliv. Rev..

[13] Hussain A.A., Starita C., Hodgetts A., Marshall J. (2010). Macromolecular diffusion characteristics of ageing human Bruch’s membrane: Implications for age-related macular degeneration (AMD). Exp. Eye Res..

[14] Cruysberg L.P.J., Nuijts R.M.M.A., Geroski D.H., Koole L.H., Hendrikse F., Edelhauser H.F. (2002). In vitro human scleral permeability of fluorescein, dexamethasone-fluorescein, methotrexate-fluorescein and rhodamine 6G and the use of a coated coil as a new drug delivery system. J. Ocul. Pharmacol. Ther..

[15] Watson P.G., Young R.D. (2004). Scleral structure, organisation and disease: A review. Exp. Eye Res..

[16] Jaffe G.J., Ashton P., Andrew P. (2006). Intraocular Drug Delivery.

[17] Ambati J., Canakis C.S., Miller J.W., Gragoudas E.S., Edwards A., Weissgold D.J., Kim I., Delori F.C., Adamis A.P. (2000). Diffusion of high molecular weight compounds through sclera. Invest. Ophthalmol. Vis. Sci..

[18] Cheruvu N.P.S., Kompella U.B. (2006). Bovine and Porcine Transscleral Solute Transport: Influence of Lipophilicity and the Choroid–Bruch’s Layer. Invest. Ophthalmol. Vis. Sci..

[19] Marsh D.A. (2011). Selection of Drug Delivery Approaches for the Back of the Eye: Opportunities and Unmet Needs. Drug Product Development for the Back of the Eye.

[20] Ashton P. (2006). Retinal Drug Delivery. Intraocular Drug Delivery.

[21] Maroñas O., García-Quintanilla L., Luaces-Rodríguez A., Fernández-Ferreiro A., Latorre-Pellicer A., Abraldes M.J., Lamas M.J., Carracedo Á. (2019). Anti-VEGF treatment and response in Age-related Macular Degeneration: Disease´s susceptibility, pharmacogenetics and pharmacokinetics. Curr. Med. Chem..

[22] García-Quintanilla L., Luaces-Rodríguez A., Gil-Martínez M., Mondelo-García C., Maroñas O., Mangas-Sanjuan V., González-Barcia M., Zarra-Ferro I., Aguiar P., Otero-Espinar F.J. (2019). Pharmacokinetics of Intravitreal Anti-VEGF Drugs in Age-Related Macular Degeneration. Pharmaceutics.

[23] Macha S., Mitra A.K. (2001). Ocular pharmacokinetics in rabbits using a novel dual probe microdialysis technique. Exp. Eye Res..

[24] Castro-Balado A., Mondelo-García C., González-Barcia M., Zarra-Ferro I., Otero-Espinar F.J., Ruibal-Morell Á., Aguiar-Fernández P., Fernández-Ferreiro A. (2019). Ocular Biodistribution Studies using Molecular Imaging. Pharmaceutics.

[25] Maurice D.M. (1997). The Regurgitation of Large Vitreous Injections. J. Ocul. Pharmacol. Ther..

[26] Stay M.S., Xu J., Randolph T.W., Barocas V.H. (2003). Computer simulation of convective and diffusive transport of controlled-release drugs in the vitreous humor. Pharm. Res..

[27] Xu J., Heys J.J., Barocas V.H., Randolph T.W. (2000). Permeability and diffusion in vitreous humor: Implications for drug delivery. Pharm. Res..

[28] Park J., Bungay P.M., Lutz R.J., Augsburger J.J., Millard R.W., Sinha Roy A., Banerjee R.K. (2005). Evaluation of coupled convective-diffusive transport of drugs administered by intravitreal injection and controlled release implant. J. Control. Release.

[29] Krishnamoorthy M.K., Park J., Augsburger J.J., Banerjee R.K. (2008). Effect of retinal permeability, diffusivity, and aqueous humor hydrodynamics on pharmacokinetics of drugs in the eye. J. Ocul. Pharmacol. Ther..

[30] Xu Q., Boylan N.J., Suk J.S., Wang Y.-Y., Nance E.A., Yang J.-C., McDonnell P.J., Cone R.A., Duh E.J., Hanes J. (2013). Nanoparticle diffusion in, and microrheology of, the bovine vitreous ex vivo. J. Control. Release.

[31] Cunha-Vaz J., Maurice D. (1969). Fluorescein dynamics in the eye. Doc. Ophthalmol..

[32] Thornit D.N., Vinten C.M., Sander B., Lund-Andersen H., la Cour M. (2010). Blood-retinal barrier glycerol permeability in diabetic macular edema and healthy eyes: Estimations from macular volume changes after peroral glycerol. Invest. Ophthalmol. Vis. Sci..

[33] Pitkänen L., Ranta V.-P., Moilanen H., Urtti A. (2005). Permeability of Retinal Pigment Epithelium: Effects of Permeant Molecular Weight and Lipophilicity. Investig. Opthalmology Vis. Sci..

[34] Käsdorf B.T., Arends F., Lieleg O. (2015). Diffusion Regulation in the Vitreous Humor. Biophys. J..

[35] Lee B., Litt M., Buchsbaum G. (1994). Rheology of the vitreous body: Part 3. Concentration of electrolytes, collagen and hyaluronic acid. Biorheology.

[36] Loukovaara S., Nurkkala H., Tamene F., Gucciardo E., Liu X., Repo P., Lehti K., Varjosalo M. (2015). Quantitative Proteomics Analysis of Vitreous Humor from Diabetic Retinopathy Patients. J. Proteome Res..

[37] Angi M., Kalirai H., Coupland S.E., Damato B.E., Semeraro F., Romano M.R. (2012). Proteomic Analyses of the Vitreous Humour. Mediat. Inflamm..

[38] Murthy K.R., Goel R., Subbannayya Y., Jacob H.K., Murthy P.R., Manda S.S., Patil A.H., Sharma R., Sahasrabuddhe N.A., Parashar A. (2014). Proteomic analysis of human vitreous humor. Clin. Proteom..

[39] Chirila T.V., Hong Y. (2016). Chapter C2 The Vitreous Humor. Handbook of Biomaterial Properties.

[40] Maurice D.M. (2012). Mishima Ocular Pharmacokinetics. Pharmacology of the Eye.

[41] Del Amo E.M., Urtti A. (2015). Rabbit as an animal model for intravitreal pharmacokinetics: Clinical predictability and quality of the published data. Exp. Eye Res..

[42] Maurice D. (2001). Review: Practical issues in intravitreal drug delivery. J. Ocul. Pharmacol. Ther..

[43] Friedrich S., Cheng Y.L., Saville B. (1997). Drug distribution in the vitreous humor of the human eye: The effects of intravitreal injection position and volume. Curr. Eye Res..

[44] Mandell B.A., Meredith T.A., Aguilar E., el-Massry A., Sawant A., Gardner S. (1993). Effects of inflammation and surgery on amikacin levels in the vitreous cavity. Am. J. Ophthalmol..

[45] Doft B.H., Weiskopf J., Nilsson-Ehle I., Wingard L.B. (1985). Amphotericin clearance in vitrectomized versus nonvitrectomized eyes. Ophthalmology.

[46] Wingard L.B., Zuravleff J.J., Doft B.H., Berk L., Rinkoff J. (1989). Intraocular distribution of intravitreally administered amphotericin B in normal and vitrectomized eyes. Invest. Ophthalmol. Vis. Sci..

[47] Ficker L., Meredith T.A., Gardner S., Wilson L.A. (1990). Cefazolin levels after intravitreal injection. Effects of inflammation and surgery. Invest. Ophthalmol. Vis. Sci..

[48] Shaarawy A., Meredith T.A., Kincaid M., Dick J., Aguilar E., Ritchie D.J., Reichley R.M. (1995). Intraocular injection of ceftazidime. Effects of inflammation and surgery. Retina.

[49] Pearson P.A., Hainsworth D.P., Ashton P. (1993). Clearance and distribution of ciprofloxacin after intravitreal injection. Retina.

[50] Aguilar H.E., Meredith T.A., el-Massry A., Shaarawy A., Kincaid M., Dick J., Ritchie D.J., Reichley R.M., Neisman M.K. (1995). Vancomycin levels after intravitreal injection. Effects of inflammation and surgery. Retina.

[51] Christoforidis J.B., Williams M.M., Wang J., Jiang A., Pratt C., Abdel-Rasoul M., Hinkle G.H., Knopp M.V. (2013). Anatomic and pharmacokinetic properties of intravitreal bevacizumab and ranibizumab after vitrectomy and lensectomy. Retina.

[52] Kakinoki M., Sawada O., Sawada T., Saishin Y., Kawamura H., Ohji M. (2012). Effect of vitrectomy on aqueous VEGF concentration and pharmacokinetics of bevacizumab in macaque monkeys. Investig. Ophthalmol. Vis. Sci..

[53] Niwa Y., Kakinoki M., Sawada T., Wang X., Ohji M. (2015). Ranibizumab and Aflibercept: Intraocular Pharmacokinetics and Their Effects on Aqueous VEGF Level in Vitrectomized and Nonvitrectomized Macaque Eyes. Investig. Ophthalmol. Vis. Sci..

[54] Edington M., Connolly J., Chong N.V. (2017). Pharmacokinetics of intravitreal anti-VEGF drugs in vitrectomized versus non-vitrectomized eyes. Expert Opin. Drug Metab. Toxicol..

[55] Peyman G.A., Vastine D.W., Raichand M. (1978). Experimental Aspects and Their Clinical Application. Ophthalmology.

[56] Campochiaro P.A., Lim J.I. (1994). Aminoglycoside toxicity in the treatment of endophthalmitis. Arch. Ophthalmol..

[57] Da M., Li K.K.W., Chan K.C., Wu E.X., Wong D.S.H. (2016). Distribution of Triamcinolone Acetonide after Intravitreal Injection into Silicone Oil-Filled Eye. BioMed Res. Int..

[58] Spitzer M.S., Kaczmarek R.T., Yoeruek E., Petermeier K., Wong D., Heimann H., Jaissle G.B., Bartz-Schmidt K.U., Szurman P. (2009). The distribution, release kinetics, and biocompatibility of triamcinolone injected and dispersed in silicone oil. Investig. Ophthalmol. Vis. Sci..

[59] Aras C., Ozdamar A., Karacorlu M., Ozkan S. (2001). Silicone oil in the surgical treatment of endophthalmitis associated with retinal detachment. Int. Ophthalmol..

[60] Mannermaa E., Vellonen K.-S., Urtti A. (2006). Drug transport in corneal epithelium and blood-retina barrier: Emerging role of transporters in ocular pharmacokinetics. Adv. Drug Deliv. Rev..

[61] Vellonen K.-S., Hellinen L., Mannermaa E., Ruponen M., Urtti A., Kidron H. (2018). Expression, activity and pharmacokinetic impact of ocular transporters. Adv. Drug Deliv. Rev..

[62] Dias C.S., Anand B.S., Mitra A.K. (2002). Effect of mono- and di-acylation on the ocular disposition of ganciclovir: Physicochemical properties, ocular bioreversion, and antiviral activity of short chain ester prodrugs. J. Pharm. Sci..

[63] Duvvuri S., Majumdar S., Mitra A.K. (2004). Role of metabolism in ocular drug delivery. Curr. Drug Metab..

[64] Dias C.S., Mitra A.K. (2000). Vitreal elimination kinetics of large molecular weight FITC-labeled dextrans in albino rabbits using a novel microsampling technique. J. Pharm. Sci..

[65] Durairaj C. (2016). Ocular Pharmacokinetics. Pharmacologic Therapy of Ocular Disease.

[66] Kwak H.W., D’Amico D.J. (1992). Evaluation of the retinal toxicity and pharmacokinetics of dexamethasone after intravitreal injection. Arch. Ophthalmol..

[67] Barza M., Lynch E., Baum J.L. (1993). Pharmacokinetics of newer cephalosporins after subconjunctival and intravitreal injection in rabbits. Arch. Ophthalmol..

[68] Robertson J.E., Westra I., Woltering E.A., Winthrop K.L., Barrie R., O’Dorisio T.M., Holmes D. (1997). Intravitreal injection of octreotide acetate. J. Ocul. Pharmacol. Ther..

[69] Leeds J.M., Henry S.P., Truong L., Zutshi A., Levin A.A., Kornbrust D. (1997). Pharmacokinetics of a potential human cytomegalovirus therapeutic, a phosphorothioate oligonucleotide, after intravitreal injection in the rabbit. Drug Metab. Dispos. Biol. Fate Chem..

[70] Durairaj C., Shah J.C., Senapati S., Kompella U.B. (2009). Prediction of vitreal half-life based on drug physicochemical properties: Quantitative structure-pharmacokinetic relationships (QSPKR). Pharm. Res..

[71] Christoforidis J.B., Chang S., Jiang A., Wang J., Cebulla C.M. (2012). Intravitreal devices for the treatment of vitreous inflammation. Mediat. Inflamm..

[72] Sides Media, www sidesmedia com Retina Today—Ocular Drug Delivery Systems for the Posterior Segment: A Review. http://retinatoday.com/2012/05/ocular-drug-delivery-systems-for-the-posterior-segment-a-review/.

[73] Shikari H., Samant P.M. (2016). Intravitreal injections: A review of pharmacological agents and techniques. J. Clin. Ophthalmol. Res..

[74] Wang J., Jiang A., Joshi M., Christoforidis J. (2013). Drug Delivery Implants in the Treatment of Vitreous Inflammation. Mediators Inflamm..

[75] Lee S.S., Hughes P., Ross A.D., Robinson M.R. (2010). Biodegradable implants for sustained drug release in the eye. Pharm. Res..

[76] Smith T.J., Pearson P.A., Blandford D.L., Brown J.D., Goins K.A., Hollins J.L., Schmeisser E.T., Glavinos P., Baldwin L.B., Ashton P. (1992). Intravitreal sustained-release ganciclovir. Arch. Ophthalmol..

[77] Abrishami M., Abrishami M., Mahmoudi A., Mosallaei N., Vakili Ahrari Roodi M., Malaekeh-Nikouei B. (2016). Solid Lipid Nanoparticles Improve the Diclofenac Availability in Vitreous after Intraocular Injection. J. Drug Deliv..

[78] Kambhampati S.P., Clunies-Ross A.J.M., Bhutto I., Mishra M.K., Edwards M., McLeod D.S., Kannan R.M., Lutty G. (2015). Systemic and Intravitreal Delivery of Dendrimers to Activated Microglia/Macrophage in Ischemia/Reperfusion Mouse Retina. Investig. Ophthalmol. Vis. Sci..

[79] Pachis K., Blazaki S., Tzatzarakis M., Klepetsanis P., Naoumidi E., Tsilimbaris M., Antimisiaris S.G. (2017). Sustained release of intravitreal flurbiprofen from a novel drug-in-liposome-in-hydrogel formulation. Eur. J. Pharm. Sci..

[80] Wang C., Seo S.-J., Kim J.-S., Lee S.-H., Jeon J.-K., Kim J.-W., Kim K.-H., Kim J.-K., Park J. (2018). Intravitreal implantable magnetic micropump for on-demand VEGFR-targeted drug delivery. J. Control. Release.

[81] (2018). Genentech: Press Releases. https://www.gene.com/media/press-releases/14739/2018-07-25/genentech-unveils-positive-phase-ii-resu.

[82] Luaces-Rodríguez A., Mondelo-García C., Zarra-Ferro I., González-Barcia M., Aguiar P., Fernández-Ferreiro A., Otero-Espinar F.J. (2020). Intravitreal anti-VEGF drug delivery systems for age-related macular degeneration. Int. J. Pharm..

[83] Barañano D.E., Kim S.J., Edelhauser H.F., Durairaj C., Kompella U.B., Handa J.T. (2009). Efficacy and pharmacokinetics of intravitreal non-steroidal anti-inflammatory drugs for intraocular inflammation. Br. J. Ophthalmol..

[84] Von Sallmann L., Meyer K., Grandi J.D. (1944). Experimental study on penicillin treatment of ectogenous infection of vitreous. Arch. Ophthalmol..

[85] Radhika M., Mithal K., Bawdekar A., Dave V., Jindal A., Relhan N., Albini T., Pathengay A., Flynn H.W. (2014). Pharmacokinetics of intravitreal antibiotics in endophthalmitis. J. Ophthalmic Inflamm. Infect..

[86] Barza M., Kane A., Baum J. (1983). Pharmacokinetics of intravitreal carbenicillin, cefazolin, and gentamicin in rhesus monkeys. Investig. Ophthalmol. Vis. Sci..

[87] Doft B.H., Barza M. (1994). Ceftazidime or Amikacin: Choice of Intravitreal Antimicrobials in the Treatment of Postoperative Endophthalmitis. Arch. Ophthalmol..

[88] Aydin E., Kazi A.A., Peyman G.A., Esfahani M.R., Muñoz-Morales A., Kivilcim M., Caro-Magdaleno M. (2007). Retinal toxicity of intravitreal doxycycline. A pilot study. Arch. Soc. Espanola Oftalmol..

[89] Iyer M.N., He F., Wensel T.G., Mieler W.F., Benz M.S., Holz E.R. (2005). Intravitreal clearance of moxifloxacin. Trans. Am. Ophthalmol. Soc..

[90] Oztürk F., Kortunay S., Kurt E., Ilker S.S., Basci N.E., Bozkurt A. (1999). Penetration of topical and oral ciprofloxacin into the aqueous and vitreous humor in inflamed eyes. Retina.

[91] Barza M., McCue M. (1983). Pharmacokinetics of aztreonam in rabbit eyes. Antimicrob. Agents Chemother..

[92] Ay G.M., Akhan S.C., Erturk S., Aktas E.S., Ozkara S.K., Caglar Y. (2004). Comparison of Intravitreal Ceftazidime and Meropenem in Treatment of Experimental Pseudomonal Posttraumatic Endophthalmitis in a Rabbit Model. J. Appl. Res..

[93] Loewenstein A., Zemel E., Lazar M., Perlman I. (1993). Drug-induced retinal toxicity in albino rabbits: The effects of imipenem and aztreonam. Investig. Ophthalmol. Vis. Sci..

[94] Conway B.P., Campochiaro P.A. (1986). Macular Infarction After Endophthalmitis Treated With Vitrectomy and Intravitreal Gentamicin. Arch. Ophthalmol..

[95] Zachary I.G., Forster R.K. (1976). Experimental Intravitreal Gentamicin. Am. J. Ophthalmol..

[96] Bakri S.J., Snyder M.R., Reid J.M., Pulido J.S., Singh R.J. (2007). Pharmacokinetics of Intravitreal Bevacizumab (Avastin). Ophthalmology.

[97] Bakri S.J., Snyder M.R., Reid J.M., Pulido J.S., Ezzat M.K., Singh R.J. (2007). Pharmacokinetics of Intravitreal Ranibizumab (Lucentis). Ophthalmology.

[98] Drug Product Development for the Back of the Eye by Uday B. Kompella, Henry F. Edelhauser | 9781441999191 | Reviews, Description and More @ BetterWorldBooks.com. https://www.betterworldbooks.com/product/detail/drug-product-development-for-the-back-of-the-eye-1441999191.

[99] Schopf L.R., Popov A.M., Enlow E.M., Bourassa J.L., Ong W.Z., Nowak P., Chen H. (2015). Topical Ocular Drug Delivery to the Back of the Eye by Mucus-Penetrating Particles. Transl. Vis. Sci. Technol..

[100] Madni A., Rahem M.A., Tahir N., Sarfraz M., Jabar A., Rehman M., Kashif P.M., Badshah S.F., Khan K.U., Santos H.A. (2017). Non-invasive strategies for targeting the posterior segment of eye. Int. J. Pharm..

[101] Ruponen M., Urtti A. (2015). Undefined role of mucus as a barrier in ocular drug delivery. Eur. J. Pharm. Biopharm..

[102] Jünemann A.G.M., Choragiewicz T., Ozimek M., Grieb P., Rejdak R. (2016). Drug bioavailability from topically applied ocular drops. Does drop size matter?. Ophthalmol. J..

[103] Vadlapudi A.D., CholKAr K., Dasari S.R., Mitra A.K. (2015). Ocular Drug Delivery.

[104] Watsky M.A., Jablonski M.M., Edelhauser H.F. (1988). Comparison of conjunctival and corneal surface areas in rabbit and human. Curr. Eye Res..

[105] Coca-Prados M. (2014). The blood-aqueous barrier in health and disease. J. Glaucoma.

[106] Barar J., Javadzadeh A.R., Omidi Y. (2008). Ocular novel drug delivery: Impacts of membranes and barriers. Expert Opin. Drug Deliv..

[107] Kaur I.P., Kakkar S. (2014). Nanotherapy for posterior eye diseases. J. Control. Release.

[108] Boddu S.H.S., Gunda S., Earla R., Mitra A.K. (2010). Ocular microdialysis: A continuous sampling technique to study pharmacokinetics and pharmacodynamics in the eye. Bioanalysis.

[109] Bravo-Osuna I., Andrés-Guerrero V., Pastoriza Abal P., Molina-Martínez I.T., Herrero-Vanrell R. (2016). Pharmaceutical microscale and nanoscale approaches for efficient treatment of ocular diseases. Drug Deliv. Transl. Res..

[110] Quantitative and Qualitative Prediction of Corneal Permeability for Drug-Like Compounds—ScienceDirect. https://www.sciencedirect.com/science/article/pii/S003991401100779X?via%3Dihub.

[111] Shen J., Deng Y., Jin X., Ping Q., Su Z., Li L. (2010). Thiolated nanostructured lipid carriers as a potential ocular drug delivery system for cyclosporine A: Improving in vivo ocular distribution. Int. J. Pharm..

[112] Sociedad Española de Oftalmología. http://www.oftalmo.com/seo/archivos/articulo.php?idSolicitud=905&numR=9&mesR=9&anioR=2001&idR=50.

[113] Ramsay E., Del Amo E.M., Toropainen E., Tengvall-Unadike U., Ranta V.-P., Urtti A., Ruponen M. (2018). Corneal and conjunctival drug permeability: Systematic comparison and pharmacokinetic impact in the eye. Eur. J. Pharm. Sci..

[114] Stjernschantz J., Selén G., Astin M., Karlsson M., Resul B. (1999). Effect of latanoprost on regional blood flow and capillary permeability in the monkey eye. Arch. Ophthalmol..

[115] Utility of Transporter/Receptor(s) in Drug Delivery to the Eye. https://www.researchgate.net/publication/236974311_Utility_of_transporterreceptors_in_drug_delivery_to_the_eye.

[116] Thrimawithana T.R., Young S., Bunt C.R., Green C., Alany R.G. (2011). Drug delivery to the posterior segment of the eye. Drug Discov. Today.

[117] Kent A.R., Nussdorf J.D., David R., Tyson F., Small D., Fellows D. (2001). Vitreous concentration of topically applied brimonidine tartrate 0.2%. Ophthalmology.

[118] Balguri S.P., Adelli G.R., Majumdar S. (2016). Topical ophthalmic lipid nanoparticle formulations (SLN, NLC) of indomethacin for delivery to the posterior segment ocular tissues. Eur. J. Pharm. Biopharm..

[119] Ying L., Tahara K., Takeuchi H. (2013). Drug delivery to the ocular posterior segment using lipid emulsion via eye drop administration: Effect of emulsion formulations and surface modification. Int. J. Pharm..

[120] Gan L., Wang J., Jiang M., Bartlett H., Ouyang D., Eperjesi F., Liu J., Gan Y. (2013). Recent advances in topical ophthalmic drug delivery with lipid-based nanocarriers. Drug Discov. Today.

[121] Davis B.M., Normando E.M., Guo L., Turner L.A., Nizari S., O’Shea P., Moss S.E., Somavarapu S., Cordeiro M.F. (2014). Topical delivery of Avastin to the posterior segment of the eye in vivo using annexin A5-associated liposomes. Small Weinh. Bergstr. Ger..

[122] Wang Y., Zheng Y., Zhang L., Wang Q., Zhang D. (2013). Stability of nanosuspensions in drug delivery. J. Control. Release.

[123] Koeberle M.J., Hughes P.M., Skellern G.G., Wilson C.G. (2006). Pharmacokinetics and disposition of memantine in the arterially perfused bovine eye. Pharm. Res..

[124] Acheampong A.A., Shackleton M., John B., Burke J., Wheeler L., Tang-Liu D. (2002). Distribution of brimonidine into anterior and posterior tissues of monkey, rabbit, and rat eyes. Drug Metab. Dispos. Biol. Fate Chem..

[125] Loftsson T., Hreinsdóttir D., Stefánsson E. (2007). Cyclodextrin microparticles for drug delivery to the posterior segment of the eye: Aqueous dexamethasone eye drops. J. Pharm. Pharmacol..

[126] Sigurdsson H.H., Konráethsdóttir F., Loftsson T., Stefánsson E. (2007). Topical and systemic absorption in delivery of dexamethasone to the anterior and posterior segments of the eye. Acta Ophthalmol. Scand..

[127] Díez J.E.B., Pujol M.M. (2002). Farmacología Ocular.

[128] Del Amo Páez E.M. (2015). Ocular and Systemic Pharmacokinetic Models for Drug Discovery and Development. Ph.D. Dissertation.

[129] Mitra A.K., Anand B.S., Duvvuri S., Fischbarg J. (2006). Drug delivery to the eye. The Biology of the Eye.

[130] Janoria K.G., Gunda S., Boddu S.H.S., Mitra A.K. (2007). Novel approaches to retinal drug delivery. Expert Opin. Drug Deliv..

[131] Hughes P., Olejnik O., Changlin J., Wilson C. (2005). Topical and systemic drug delivery to the posterior segments. Adv. Drug Deliv. Rev..

[132] Kwan A.S.L., Barry C., McAllister I.L., Constable I. (2006). Fluorescein angiography and adverse drug reactions revisited: The Lions Eye experience. Clin. Exp. Ophthalmol..

[133] Vellonen K.-S., Soini E.-M., del Amo E.M., Urtti A. (2016). Prediction of Ocular Drug Distribution from Systemic Blood Circulation. Mol. Pharm..

[134] Hosoya K., Tomi M. (2005). Advances in the cell biology of transport via the inner blood-retinal barrier: Establishment of cell lines and transport functions. Biol. Pharm. Bull..

[135] Routes of Administration for Ocular Medications—Pharmacology. https://www.msdvetmanual.com/pharmacology/systemic-pharmacotherapeutics-of-the-eye/routes-of-administration-for-ocular-medications.

[136] Gkretsi V., Zacharia L.C., Stylianopoulos T. (2017). Targeting inflammation to improve tumor drug delivery. Trends Cancer.

[137] Urtti A. (2006). Challenges and obstacles of ocular pharmacokinetics and drug delivery. Adv. Drug Deliv. Rev..

[138] Toda R., Kawazu K., Oyabu M., Miyazaki T., Kiuchi Y. (2011). Comparison of Drug Permeabilities across the Blood–Retinal Barrier, Blood–Aqueous Humor Barrier, and Blood–Brain Barrier. J. Pharm. Sci..

[139] Gaudana R., Jwala J., Boddu S.H.S., Mitra A.K. (2009). Recent Perspectives in Ocular Drug Delivery. Pharm. Res..

[140] Jiye Y., Jianting Z. (2011). Multidrug resistance-associated protein 1 (MRP1/ABCC1) polymorphism: From discovery to clinical application. Zhong Nan Da Xue Xue Bao Yi Xue Ban.

[141] ABCC1 Gene—GeneCards | MRP1 Protein | MRP1 Antibody. https://www.genecards.org/cgi-bin/carddisp.pl?gene=ABCC1.

[142] Pitkänen L., Ranta V.-P., Moilanen H., Urtti A. (2007). Binding of Betaxolol, Metoprolol and Oligonucleotides to Synthetic and Bovine Ocular Melanin, and Prediction of Drug Binding to Melanin in Human Choroid-Retinal Pigment Epithelium. Pharm. Res..

[143] Demetriades A.M., Deering T., Liu H., Lu L., Gehlbach P., Packer J.D., Gabhann F.M., Popel A.S., Wei L.L., Campochiaro P.A. (2008). Trans-scleral Delivery of Antiangiogenic Proteins. J. Ocul. Pharmacol. Ther..

[144] Menon I.A., Wakeham D.C., Persad S.D., Avaria M., Trope G.E., Basu P.K. (1992). Quantitative determination of the melanin contents in ocular tissues from human blue and brown eyes. J. Ocul. Pharmacol. Ther..

[145] Schoenwald R.D., Tandon V., Wurster D.E., Barfknecht C.F. (1998). Significance of melanin binding and metabolism in the activity of 5-acetoxyacetylimino-4-methyl-Δ2-1, 3, 4,-thiadiazoline-2-sulfonamide1. Eur. J. Pharm. Biopharm..

[146] Leblanc B., Jezequel S., Davies T., Hanton G., Taradach C. (1998). Binding of drugs to eye melanin is not predictive of ocular toxicity. Regul. Toxicol. Pharmacol..

[147] Larsson B.S. (1993). Interaction between chemicals and melanin. Pigment Cell Res..

[148] Bill A., Törnquist P., Alm A. (1980). Permeability of the intraocular blood vessels. Trans. Ophthalmol. Soc..

[149] Guymer R.H., Bird A.C., Hageman G.S. (2004). Cytoarchitecture of Choroidal Capillary Endothelial Cells. Investig. Opthalmol. Vis. Sci..

[150] Ranta V.-P., Mannermaa E., Lummepuro K., Subrizi A., Laukkanen A., Antopolsky M., Murtomäki L., Hornof M., Urtti A. (2010). Barrier analysis of periocular drug delivery to the posterior segment. J. Control. Release.

[151] Patel A. (2013). Ocular drug delivery systems: An overview. World J. Pharmacol..

[152] Constable P.A., Lawrenson J.G., Dolman D.E.M., Arden G.B., Abbott N.J. (2006). P-Glycoprotein expression in human retinal pigment epithelium cell lines. Exp. Eye Res..

[153] Eye Drugs—Prescribing and Administering. Patient. https://patient.info/doctor/eye-drugs-prescribing-and-administering.

[154] Samtani S., Amaral J., Campos M.M., Fariss R.N., Becerra S.P. (2009). Doxycycline-Mediated Inhibition of Choroidal Neovascularization. Investig. Opthalmol. Vis. Sci..

[155] Kampougeris G. (2005). Penetration of moxifloxacin into the human aqueous humour after oral administration. Br. J. Ophthalmol..

[156] García D.A.R., Santos Garcia A. (2011). Chapter 5 Quimioterapia inmunosupresora en uveítis. Oftalmología en la Opinión de los Expertos.

[157] Kaur I.P., Smitha R., Aggarwal D., Kapil M. (2002). Acetazolamide: Future perspective in topical glaucoma therapeutics. Int. J. Pharm..

[158] Shirasaki Y. (2008). Molecular Design for Enhancement of Ocular Penetration. J. Pharm. Sci..

[159] Pérez-Blázquez E., Redondo M.I., Gracia T. (2008). Sida y oftalmología: Una visión actual. An. Sist. Sanit. Navar..

[160] Benatar-Haserfaty J., Flores J.A.P. (2003). Anestesia locorregional en oftalmología: Una puesta al día. Oculoplastia.

[161] García E., Mensa J., Martínez J.A. (2001). Diffusion and pharmacokinetics of antibiotics in the ocular globus. Therapeutic implications. Rev. Esp. Quim..

[162] Lin P., Suhler E.B., Rosenbaum J.T. (2014). The Future of Uveitis Treatment. Ophthalmology.

[163] Duica I., Voinea L.-M., Mitulescu C., Istrate S., Coman I.-C., Ciuluvica R. (2018). The use of biologic therapies in uveitis. Rom. J. Ophthalmol..

[164] Kahn M. (2005). Bioavailability of vitamin B using a small-volume nebulizer ophthalmic drug delivery system. Clin. Exp. Ophthalmol..

[165] Yoo W.S., Kim C.R., Kim B.J., Ahn S.K., Seo S.W., Yoo J.M., Kim S.J. (2015). Successful Treatment of Infectious Scleritis by Pseudomonas aeruginosa with Autologous Perichondrium Graft of Conchal Cartilage. Yonsei Med. J..

[166] Schwartz S.G., Flynn H.W. (2014). Update on the prevention and treatment of endophthalmitis. Expert Rev. Ophthalmol..

[167] Sallam A.B., Kirkland K.A., Barry R., Soliman M.K., Ali T.K., Lightman S. (2018). A Review of Antimicrobial Therapy for Infectious Uveitis of the Posterior Segment. Med. Hypothesis Discov. Innov. Ophthalmol..

[168] Unidad de Enfermedades Vitreorretinianas—FISABIO. http://fisabio.san.gva.es/unidad-de-enfermedades-vitreorretinianas1.

[169] Andrés S., Higueras M.I., Mozaz T. (2008). Efectos Adversos Oculares Asociados a Medicamentos y Productos Oftálmicos. Colegio Oficial de Farmacéuticos de Zaragoza. Vocalía de Optica. https://www.academiadefarmaciadearagon.es/docs/Documentos/Documento24.pdf.

[170] Kim H., Robinson M.R., Lizak M.J., Tansey G., Lutz R.J., Yuan P., Wang N.S., Csaky K.G. (2004). Controlled Drug Release from an Ocular Implant: An Evaluation Using Dynamic Three-Dimensional Magnetic Resonance Imaging. Investig. Opthalmology Vis. Sci..

[171] Geroski D.H., Edelhauser H.F. (2000). Drug delivery for posterior segment eye disease. Investig. Ophthalmol. Vis. Sci..

[172] Prausnitz M.R., Noonan J.S. (1998). Permeability of cornea, sclera, and conjunctiva: A literature analysis for drug delivery to the eye. J. Pharm. Sci..

[173] Conrad J.M., Robinson J.R. (1980). Mechanisms of anterior segment absorption of pilocarpine following subconjunctival injection in albino rabbits. J. Pharm. Sci..

[174] Kaiser P.K., Goldberg M.F., Davis A.A. (2007). Posterior Juxtascleral Depot Administration of Anecortave Acetate. Surv. Ophthalmol..

[175] Ambati J., Adamis A.P. (2002). Transscleral drug delivery to the retina and choroid. Prog. Retin. Eye Res..

[176] Ghate D., Edelhauser H.F. (2006). Ocular drug delivery. Expert Opin. Drug Deliv..

[177] Lee S.-B., Geroski D.H., Prausnitz M.R., Edelhauser H.F. (2004). Drug delivery through the sclera: Effects of thickness, hydration, and sustained release systems. Exp. Eye Res..

[178] Bourges J.L., Bloquel C., Thomas A., Froussart F., Bochot A., Azan F., Gurny R., BenEzra D., Behar-Cohen F. (2006). Intraocular implants for extended drug delivery: Therapeutic applications. Adv. Drug Deliv. Rev..

[179] Raghava S., Hammond M., Kompella U.B. (2004). Periocular routes for retinal drug delivery. Expert Opin. Drug Deliv..

[180] Olsen T.W., Edelhauser H.F., Lim J.I., Geroski D.H. (1995). Human scleral permeability. Effects of age, cryotherapy, transscleral diode laser, and surgical thinning. Investig. Ophthalmol. Vis. Sci..

[181] Ambati J., Gragoudas E.S., Miller J.W., You T.T., Miyamoto K., Delori F.C., Adamis A.P. (2000). Transscleral delivery of bioactive protein to the choroid and retina. Investig. Ophthalmol. Vis. Sci..

[182] Marmor M.F., Negi A., Maurice D.M. (1985). Kinetics of macromolecules injected into the subretinal space. Exp. Eye Res..

[183] Geroski D.H., Edelhauser H.F. (2001). Transscleral drug delivery for posterior segment disease. Adv. Drug Deliv. Rev..

[184] Kim S.H., Csaky K.G., Wang N.S., Lutz R.J. (2008). Drug elimination kinetics following subconjunctival injection using dynamic contrast-enhanced magnetic resonance imaging. Pharm. Res..

[185] Guo W., Zhu Y., Yu P.K., Yu X., Sun X., Cringle S.J., Su E.-N., Yu D.-Y. (2012). Quantitative study of the topographic distribution of conjunctival lymphatic vessels in the monkey. Exp. Eye Res..

[186] Myles M.E., Neumann D.M., Hill J.M. (2005). Recent progress in ocular drug delivery for posterior segment disease: Emphasis on transscleral iontophoresis. Adv. Drug Deliv. Rev..

[187] Shah S.S., Denham L.V., Elison J.R., Bhattacharjee P.S., Clement C., Huq T., Hill J.M. (2010). Drug delivery to the posterior segment of the eye for pharmacologic therapy. Expert Rev. Ophthalmol..

[188] Kompella U.B., Bandi N., Ayalasomayajula S.P. (2003). Subconjunctival nano- and microparticles sustain retinal delivery of budesonide, a corticosteroid capable of inhibiting VEGF expression. Investig. Ophthalmol. Vis. Sci..

[189] Ayalasomayajula S.P., Kompella U.B. (2003). Celecoxib, a selective cyclooxygenase-2 inhibitor, inhibits retinal vascular endothelial growth factor expression and vascular leakage in a streptozotocin-induced diabetic rat model. Eur. J. Pharmacol..

[190] Ayalasomayajula S.P., Kompella U.B. (2004). Retinal delivery of celecoxib is several-fold higher following subconjunctival administration compared to systemic administration. Pharm. Res..

[191] Ayalasomayajula S.P., Kompella U.B. (2005). Subconjunctivally administered celecoxib-PLGA microparticles sustain retinal drug levels and alleviate diabetes-induced oxidative stress in a rat model. Eur. J. Pharmacol..

[192] Amrite A.C., Ayalasomayajula S.P., Cheruvu N.P.S., Kompella U.B. (2006). Single periocular injection of celecoxib-PLGA microparticles inhibits diabetes-induced elevations in retinal PGE2, VEGF, and vascular leakage. Investig. Ophthalmol. Vis. Sci..

[193] Misra G.P., Singh R.S.J., Aleman T.S., Jacobson S.G., Gardner T.W., Lowe T.L. (2009). Subconjunctivally implantable hydrogels with degradable and thermoresponsive properties for sustained release of insulin to the retina. Biomaterials.

[194] Tsui J.Y., Dalgard C., Van Quill K.R., Lee L., Grossniklaus H.E., Edelhauser H.F., O’Brien J.M. (2008). Subconjunctival topotecan in fibrin sealant in the treatment of transgenic murine retinoblastoma. Investig. Ophthalmol. Vis. Sci..

[195] Gangaputra S., Newcomb C.W., Liesegang T.L., Kaçmaz R.O., Jabs D.A., Levy-Clarke G.A., Nussenblatt R.B., Rosenbaum J.T., Suhler E.B., Thorne J.E. (2009). Methotrexate for Ocular Inflammatory Diseases. Ophthalmology.

[196] Wong C.W., Czarny B., Metselaar J.M., Ho C., Ng S.R., Barathi A.V., Storm G., Wong T.T. (2018). Evaluation of subconjunctival liposomal steroids for the treatment of experimental uveitis. Sci. Rep..

[197] Rowe-Rendleman C.L., Durazo S.A., Kompella U.B., Rittenhouse K.D., Di Polo A., Weiner A.L., Grossniklaus H.E., Naash M.I., Lewin A.S., Horsager A. (2014). Drug and Gene Delivery to the Back of the Eye: From Bench to Bedside. Investig. Ophthalmol. Vis. Sci..

[198] Imai H., Misra G.P., Wu L., Janagam D.R., Gardner T.W., Lowe T.L. (2015). Subconjunctivally Implanted Hydrogels for Sustained Insulin Release to Reduce Retinal Cell Apoptosis in Diabetic Rats. Investig. Ophthalmol. Vis. Sci..

[199] Ghate D., Brooks W., McCarey B.E., Edelhauser H.F. (2007). Pharmacokinetics of intraocular drug delivery by periocular injections using ocular fluorophotometry. Investig. Ophthalmol. Vis. Sci..

[200] Roper-Hall M.J. (1989). Anesthesia and Akinesia for Eye Operations.

[201] Canavan K.S., Dark A., Garrioch M.A. (2003). Sub-Tenon’s administration of local anaesthetic: A review of the technique. Br. J. Anaesth..

[202] JJ K., Bowling B. (2011). Clinical Ophthalmology: A Systematic Approach.

[203] Ehlers J.P., Gregory L.F. (2008). The Wills Eye Manual: Office and Emergency Room Diagnosis and Treatment of Eye Disease.

[204] Lafranco Dafflon M., Tran V.T., Guex-Crosier Y., Herbort C.P. (1999). Posterior sub-Tenon’s steroid injections for the treatment of posterior ocular inflammation: Indications, efficacy and side effects. Graefes Arch. Clin. Exp. Ophthalmol..

[205] Tanner V., Kanski J.J., Frith P.A. (1998). Posterior sub-Tenon’s triamcinolone injections in the treatment of uveitis. Eye Lond. Engl..

[206] Choi Y.J., Oh I.K., Oh J.R., Huh K. (2006). Intravitreal versus posterior subtenon injection of triamcinolone acetonide for diabetic macular edema. Korean J. Ophthalmol. KJO.

[207] Cardillo J.A., Melo L.A.S., Costa R.A., Skaf M., Belfort R., Souza-Filho A.A., Farah M.E., Kuppermann B.D. (2005). Comparison of intravitreal versus posterior sub-Tenon’s capsule injection of triamcinolone acetonide for diffuse diabetic macular edema. Ophthalmology.

[208] Ozkiriş A., Erkiliç K. (2005). Complications of intravitreal injection of triamcinolone acetonide. Can. J. Ophthalmol..

[209] Shen L., You Y., Sun S., Chen Y., Qu J., Cheng L. (2010). Intraocular and systemic pharmacokinetics of triamcinolone acetonide after a single 40-mg posterior subtenon application. Ophthalmology.

[210] Accola P.J., Bentley E., Smith L.J., Forrest L.J., Baumel C.A., Murphy C.J. (2006). Development of a retrobulbar injection technique for ocular surgery and analgesia in dogs. J. Am. Vet. Med. Assoc..

[211] Kazancioglu L., Batcik S., Kazdal H., Sen A., Sekeryapan Gediz B., Erdivanli B. (2017). Complication of Peribulbar Block: Brainstem Anaesthesia. Turk. J. Anesth. Reanim..

[212] Mehta S., Laird P., Debiec M., Hwang C., Zhang R., Yan J., Hendrick A., Hubbard G.B., Bergstrom C.S., Yeh S. (2018). Formulation of a Peribulbar Block for Prolonged Postoperative Pain Management in Vitreoretinal Surgery. Ophthalmol. Retina.

[213] Iriyama A., Obata R., Inoue Y., Takahashi H., Tamaki Y., Yanagi Y. (2008). Effect of posterior juxtascleral triamcinolone acetonide on the efficacy and choriocapillaris hypoperfusion of photodynamic therapy. Graefes Arch. Clin. Exp. Ophthalmol..

[214] Hayek S., Scherrer M., Barthelmes D., Fleischhauer J., Kurz-Levin M., Menghini M., Helbig H., Sutter F. (2007). First Clinical Experience with Anecortave Acetate (Retaane^®^). Klin. Monatsblätter Für Augenheilkd..

[215] Patel S.R., Lin A.S.P., Edelhauser H.F., Prausnitz M.R. (2011). Suprachoroidal Drug Delivery to the Back of the Eye Using Hollow Microneedles. Pharm. Res..

[216] Krohn J., Bertelsen T. (2009). Corrosion casts of the suprachoroidal space and uveoscleral drainage routes in the human eye. Acta Ophthalmol. Scand..

[217] Krohn J., Bertelsen T. (1998). Light microscopy of uveoscleral drainage routes after gelatine injections into the suprachoroidal space. Acta Ophthalmol. Scand..

[218] Einmahl S., Savoldelli M., D’Hermies F., Tabatabay C., Gurny R., Behar-Cohen F. (2002). Evaluation of a Novel Biomaterial in the Suprachoroidal Space of the Rabbit Eye. Retina.

[219] Olsen T.W., Feng X., Wabner K., Conston S.R., Sierra D.H., Folden D.V., Smith M.E., Cameron J.D. (2006). Cannulation of the Suprachoroidal Space: A Novel Drug Delivery Methodology to the Posterior Segment. Am. J. Ophthalmol..

[220] Kompella U.B., Edelhauser H.F., American Association of Pharmaceutical Scientists (2011). Drug Product Development for the Back of the Eye.

[221] Liu Suprachoroidal Injection of Ketorolac Tromethamine does not Cause Retinal Damage. http://www.nrronline.org/article.asp?issn=1673-5374;year=2012;volume=7;issue=35;spage=2770;epage=2777;aulast=Liu.

[222] Patel S.R., Berezovsky D.E., McCarey B.E., Zarnitsyn V., Edelhauser H.F., Prausnitz M.R. (2012). Targeted Administration into the Suprachoroidal Space Using a Microneedle for Drug Delivery to the Posterior Segment of the Eye. Investig. Opthalmol. Vis. Sci..

[223] Falavarjani K.G., Nguyen Q.D. (2013). Adverse events and complications associated with intravitreal injection of anti-VEGF agents: A review of literature. Eye.

[224] Peng Y., Tang L., Zhou Y. (2017). Subretinal Injection: A Review on the Novel Route of Therapeutic Delivery for Vitreoretinal Diseases. Ophthalmic Res..

[225] Johnson C.J., Berglin L., Chrenek M.A., Redmond T.M., Boatright J.H., Nickerson J.M. (2008). Technical brief: Subretinal injection and electroporation into adult mouse eyes. Mol. Vis..

[226] Timmers A.M., Zhang H., Squitieri A., Gonzalez-Pola C. (2001). Subretinal injections in rodent eyes: Effects on electrophysiology and histology of rat retina. Mol. Vis..

[227] Qi Y., Dai X., Zhang H., He Y., Zhang Y., Han J., Zhu P., Zhang Y., Zheng Q., Li X. (2015). Trans-Corneal Subretinal Injection in Mice and Its Effect on the Function and Morphology of the Retina. PLoS ONE.

